# Electron‐Rich Heptacyclic S,N Heteroacene Enabling C‐Shaped A‐D‐A‐type Electron Acceptors With Photoelectric Response beyond 1000 Nm for Highly Sensitive Near‐Infrared Photodetectors

**DOI:** 10.1002/advs.202413045

**Published:** 2025-01-14

**Authors:** Kuo‐Hsiu Huang, Bing‐Huang Jiang, Han‐Cheng Lu, Yung‐Jing Xue, Chia‐Fang Lu, Yung‐Yung Chang, Ching‐Li Huang, Su‐Ying Chien, Chih‐Ping Chen, Yen‐Ju Cheng

**Affiliations:** ^1^ Department of Applied Chemistry National Yang Ming Chiao Tung University Hsinchu 30010 Taiwan; ^2^ Department of Materials Engineering Ming Chi University of Technology New Taipei City 243303 Taiwan; ^3^ Instrumentation Center National Taiwan University Taipei 10617 Taiwan; ^4^ College of Engineering and Center for Sustainability and Energy Technologies Chang Gung University Taoyuan 33302 Taiwan; ^5^ Center for Emergent Functional Matter Science National Yang Ming Chiao Tung University Hsinchu 30010 Taiwan

**Keywords:** electron acceptors, heteroacene, near‐infrared absorption, organic photodetectors, organic photovoltaics

## Abstract

A highly electron‐rich S,N heteroacene building block is developed and condensed with FIC and Cl‐IC acceptors to furnish CT‐F and CT‐Cl, which exhibit near‐infrared (NIR) absorption beyond 1000 nm. The C‐shaped CT‐F and CT‐Cl self‐assemble into a highly ordered 3D intermolecular packing network via multiple π−π interactions in the single crystal structures. The CT‐F‐based organic photovoltaic (OPV) achieved an impressive efficiency of 14.30% with a broad external quantum efficiency response extending from the UV‐vis to the NIR (300–1050 nm) regions, outperforming most binary OPVs employing NIR A‐D‐A‐type acceptors. CT‐Cl possesses a higher surface energy than CT‐F, promoting vertical phase segregation and resulting in its preferential accumulation near the bottom interface of the blend. This arrangement, combined with the lower HOMO energy level of CT‐Cl, effectively reduces undesired hole and electron injection under reverse voltage. The PM6:CT‐Cl‐based organic photodetectors (OPDs) devices achieved an ultra‐high shot‐noise‐limited specific detectivity (*D*_sh_*) values exceeding 10^14^ Jones in the NIR region from 620 to 1000 nm, reaching an unprecedentedly high value of 1.3 × 10^14^ Jones at 950 nm. When utilizing a 780 nm light source, the PM6:CT‐Cl‐based OPDs show record‐high rise/fall times of 0.33/0.11 µs and an exceptional cut‐off frequency (*f*_‐3dB_) of 590 kHz at −1 V.

## Graphical abstract

C‐shaped thienodipyrrole‐based A‐D‐A acceptors, CT‐F and CT‐Cl, exhibiting near‐infrared (NIR) absorption beyond 1000 nm, have been designed and synthesized. The CT‐F‐based organic photovoltaic (OPV) achieves a PCE of 14.30%, representing the highest value for OPVs with photoresponses exceeding 1000 nm. The PM6:CT‐Cl organic photodetector (OPD) device, featuring more pronounced vertical phase separation to effectively inhibit the unfavorable charge injections, achieved a low *J*_d_ of 2.1 × 10⁻¹¹ A cm⁻^2^ and an ultra‐high detectivity *D*_sh_* of 1.3 × 10¹⁴ Jones.

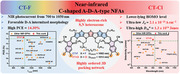

## 1 Introduction

Organic semiconductors possessing photoelectric response characteristics in the near‐infrared (NIR) region have attracted considerable research interest for their potential in high‐performance organic photovoltaics (OPVs) and NIR organic photodetectors (OPDs).^[^ ^1^ ^]^ Considering that ≈50% of solar radiation energy lies within the NIR spectrum, the design strategy for organic materials in OPV development is increasingly focusing on extending light absorption into the NIR region to enhance overall device performance. The organic materials with absorption extending to or exceeding 1000 nm hold significant importance for NIR OPDs in applications such as biological imaging, optical communication, sensing and nocturnal surveillance. The breakthrough of OPVs research occurred with the emergence of non‐fullerene organic n‐type acceptors (NFAs) on the basis of A‐D‐A architectures enabling the straightforward adjustment of their absorption characteristics through chemical structure modifications. Up to now, most A‐D‐A fused‐ring electron acceptors exhibit NIR absorption below 900 nm. Numerous multi‐fused rings such as thieno[3,2‐*b*]thiophene (TT),^[^ ^2^ ^]^ benzo‐[1,2‐*b*:4,5‐*b*′]dithiophene (BDT),^[^ ^3^ ^]^ dithieno[3,2‐*b*]thiophenecyclopentacarbazole (DTTC),^[^ ^4^ ^]^ 4,8‐dihydropentaleno[1,2‐b:4,5‐*b*′]dithiophene (PDT),^[^ ^5^ ^]^ and indacenodithieno[3,2‐*b*]thiophene (IDTT),^[^ ^6^ ^]^ have been utilized as the electron‐rich central D cores which are further end‐capped through condensation with various electron‐withdrawing end groups. By enhancing the electron‐donating strength of multifused ring D to facilitate the photo‐induced intramolecular charge transfer (ICT) in the materials, absorption of the resulting NFAs can extend beyond 900 nm in the solid state. The low‐bandgap A‐D‐A acceptor materials combined with wide‐bandgap donor materials can be utilized in tandem and ternary blend OPVs to optimize the UV–vis‐NIR light‐harvesting efficiency, thereby maximizing the short‐circuit current (*J*_sc_).^[^ ^7^ ^]^ The NIR‐absorbing materials can also be applied in semi‐transparent OPVs designed for use in greenhouses where specific wavelengths can assist in promoting plant growth.^[^ ^8^ ^]^ The success of NIR electron acceptors in OPVs has simultaneously expedited the development of NIR organic photodetectors (OPDs).^[^ ^5^, ^9^ ^]^ Nonetheless, binary OPV devices that incorporate NIR NFAs with narrow bandgaps often yield comparatively lower device efficiency due to the larger energy loss.^[^ ^7^, ^10^ ^]^ NIR NFAs also have difficulty in suppressing charge injection under reverse biases, resulting in elevated dark current in OPDs. A more critical challenge is that NFAs with absorption beyond 1000 nm remain rare due to the limited availability of strong electron‐rich cores. To date, the most successful NFA, known as Y6, features an A‐D_N_A′_N_D‐A structure where the D_N_A′_N_D is a heptacyclic ladder‐type structure and A is the 2‐(5,6‐difluoro‐3‐oxo‐2,3‐dihydro‐1*H*‐inded‐1‐ylidene)malononitrile (FIC) end‐group. The OPV binary devices using Y6‐series NFAs reached power conversion efficiencies (PCEs) of 15%–19%.^[^ ^11^ ^]^ The D_N_A′_N_D contains a central benzothiadizole unit (A′) which is fused with two thieno[3,2‐*b*]thiophenes (TT, D) by nitrogen bridges (subscript N). The *C*_2v_‐symmetric and C‐shaped A‐D_N_A′_N_D‐A architecture enables the formation of 3D elliptical packing networks through multiple π—π interactions, which is believed to play a crucial role in achieving the superior OPV performance.^[^ ^12^ ^]^ Y6‐based derivatives blended with polymers have also been widely applied for organic photodetectors.^[^ ^9^, ^13^ ^]^ Nevertheless, the electron‐accepting benzothiadizole A′ unit induces an intramolecular charge transfer (ICT) within the D_N_A′_N_D structure, which subsequently diminishes the primary ICT from the D_N_A′_N_D to the A moieties laterally.^[^ ^14^ ^]^ Therefore, the absorption onsets of the Y6‐series NFAs in thin‐film state are limited to below 950 nm, hindering their suitability for NIR II (900–1700 nm) applications.

To design a new NFA capable of absorbing light beyond 1000 nm while preserving the essential C‐shaped molecular geometry similar to the Y6‐series NFAs, a straightforward approach is to replace the central benzothiadiazole A′ unit in D_N_A′_N_D with an electron‐rich thiophene (T). This modification yields a heptacyclic S,N heteroacene symbolized as TT_N_T_N_TT having a strong electron‐donating ability compared to typical carbon‐bridged multifused rings.

**Figure 1:**
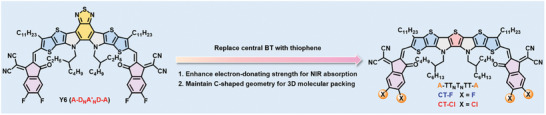
The chemical structures of Y6 and the C‐shaped A‐TT_N_T_N_TT‐A‐type electron acceptors, CT‐F and CT‐Cl, used in this research.

Utilizing TT_N_T_N_TT as the central heteroarene donor core in the design of C‐shaped A‐D‐A‐type NFAs enhances the ICT effect and strengthens π−π stacking interactions, thereby improving NIR light‐harvesting capabilities while preserving orderly molecular packing. Surprisingly, C‐shaped A‐TT_N_T_N_TT‐A‐type electron acceptors have yet to be explored (**Figure** **1**).

We herein developed a highly electron‐rich thienodipyrrole‐based TT_N_T_N_TT building block, synthesized by palladium‐catalyzed Negishi coupling and Buchwald‐Hartwig amination. Diformylated TT_N_T_N_TT was subsequently coupled with FIC or Cl‐IC acceptors to furnish two NIR electron acceptors referred to as CT‐F and CT‐Cl (for simple nomenclature, C stands for C‐shape, T stands for the central thiophene, and F/Cl stand for FIC and Cl‐IC acceptors, respectively). The two materials exhibiting NIR absorption beyond 1000 nm were combined with the wide bandgap polymer PM6 for OPVs and OPDs. The CT‐F‐based binary device has achieved an impressive PCE of 14.30% with a short‐circuit current (*J*_sc_) of 27.21 mA cm^2^, and a fill factor (FF) of 67.6%. This outcome represents the highest efficiency reported for NIR binary OPVs employing A‐D‐A‐type acceptors, with an optoelectronic response over 1000 nm. The CT‐Cl‐based device also demonstrated significant NIR responsiveness, making it highly suitable for applications in NIR organic photodetectors (NIR‐OPDs). Under optimal conditions, the PM6:CT‐Cl‐based OPD achieved ultra‐high shot‐noise‐limited specific detectivity (*D*_sh_*) values exceeding 10^14^ Jones in the NIR region from 620 to 1000 nm with an unprecedentedly high value of 1.3 × 10^14^ Jones at 950 nm. These results surpass the performance of most organic‐based NIR photodiode‐type OPDs reported to date.

## 2 Results and Discussion

### 2.1 Synthesis of the NIR Materials

**Scheme 1:**
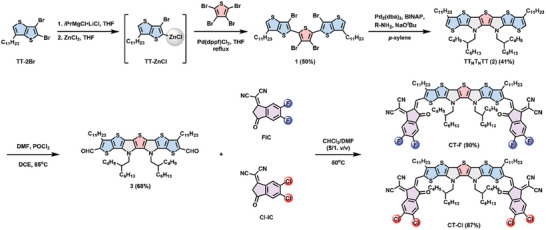
Synthetic protocol for the A‐TT_N_T_N_TT‐A‐type NFAs, CT‐F and CT‐Cl.

The synthetic route for CT‐F and CT‐Cl is illustrated in **Scheme** **1**. TT‐2Br was synthesized according to the procedures reported in the literature.^[^ ^12^ ^]^ The Grignard metathesis of TT‐2Br selectively at the α‐position of the thiophene, followed by quenching with ZnCl_2_, resulted in the formation of a TT‐ZnCl intermediate. Palladium‐catalyzed Negishi coupling of TT‐ZnCl with 2,3,4,5‐tetrabromothiophene regioselectively occurred at the 2,5‐positions of the thiophene to furnish compound **1**. Palladium‐catalyzed Buchwald‐Hartwig amination of compound **1** with 2‐butyloctylamine led to the formation of two pyrrole rings embedded in the heptacyclic ladder structure **2** (TT_N_T_N_TT). Formylation of **2** by the Vilsmeier reaction (DMF/POCl_3_) afforded **3** which was further subjected to Knoevenagel condensation with the FIC and Cl‐IC acceptors to afford CT‐F and CT‐Cl in 90% and 87% yields, respectively.

### 2.2 Thermal Properties

**Figure 2:**
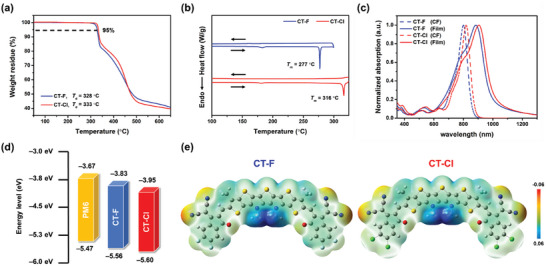
a) TGA and b) DSC measurements of CT‐F and CT‐Cl with a ramping rate of 10 °C min^−1^. c) Normalized absorption spectra of CT‐F and CT‐Cl in chloroform (CF) solution and thin films. d) HOMO/LUMO energy levels diagram of PM6, CT‐F and CT‐Cl. e) Electrostatic potential (ESP) distributions of CT‐F and CT‐Cl calculated by DFT at B3LYP/6‐311G(d,p) level.

Thermogravimetric analysis (TGA) and differential scanning calorimetry (DSC) were performed to examine the thermal characteristics of the materials shown in **Figure** **2**a,b. CT‐Cl demonstrates a higher decomposition temperature (*T*_d_) of 333 °C than CT‐F with the *T*_d_ value of 328 °C, indicating that the introduction of chlorine atoms marginally improves its thermal stability. DSC measurements reveal that CT‐Cl shows a much higher melting point (*T*_m_) of 316 °C compared to CT‐F with a *T*_m_ of 277 °C upon heating. This indicates that the presence of chlorine atoms enhances intermolecular interactions. The peaks observed at 183 °C for CT‐F and 182 °C for CT‐Cl are likely be attributed to their glass transition temperatures (*T*_g_). These values are comparable to those reported for ITIC derivatives.^[^ ^15^ ^]^

### 2.3 Optical and Electrochemical Properties

Absorption spectra of CT‐F and CT‐Cl are illustrated in Figure 2c, and the optical properties are summarized in **Table** **1**. Compared to the Y6 counterpart, both NFAs display absorption bands extending to NIR region from 750 to 900 nm in a chloroform solution with absorption maxima (λ_max_) located at 800 nm for CT‐F and 818 nm for CT‐Cl, respectively. CT‐F and CT‐Cl show notable band broadening and bathochromic shift in the film state. The absorption λ_max_ and onsets for CT‐F and CT‐Cl dramatically extend to 886/1002 nm and 909/1013 nm (821/931 nm for Y6), respectively, suggesting strong intermolecular π–π interactions and compact molecular packing in the solid state. From the onset of the thin film absorption, the optical band gaps of A‐TT_N_T_N_TT‐A‐based CT‐F and CT‐Cl are estimated to be 1.24 and 1.22 eV, respectively. These values are significantly smaller than that of the typical A‐D_N_A'_N_D‐A Y6 (1.33 eV), indicating that TT_N_T_N_TT acts as a much stronger electron donor to strengthen the ICT effect compared to D_N_A'_N_D. Furthermore, replacing fluorine with chlorine in the end group further narrows the bandgap due to the increased dipole moment and increased crystallinity of CT‐Cl.^[^ ^16^ ^]^

**Table 1:** Optical and electrochemical properties of CT‐F and CT‐Cl.

NFAs	Extinction coefficient [× 10^5^ cm^−1^ M^−1^]^a^ ^)^	λ_max_ [nm]		∆λ [nm]	λ_onset_ [nm]^b^ ^)^	*E*_g_^opt^ [eV] ^c^ ^)^	HOMO [eV]^d^ ^)^	LUMO [eV]^d^ ^)^	*E*_g_^ele^ [eV]^d^ ^)^
		CF	Film						
CT‐F	1.9	800	886	86	1002	1.24	−5.56	−3.83	1.73
CT‐Cl	2.8	818	909	91	1013	1.22	−5.60	−3.95	1.65

^a)^ Calculated at λ_max_ in solution state;

^b)^ Absorption edge of the thin film;

^c)^ *E*_g_^opt^ = 1240/λ_onset_;

^d)^ Determined by cyclic voltammetry.

Cyclic voltammetry (CV) was employed to examine the electrochemical characteristics of the materials (Figure 2d and Table 1 and Figure S1, Supporting Information). The highest occupied molecular orbital and lowest unoccupied molecular orbital (HOMO/LUMO) energy levels for CT‐F and CT‐Cl are estimated to be −5.56/−3.83 eV and −5.60/−3.95 eV, respectively. The corresponding *E*_g_^CV^ values are calculated to be 1.73 and 1.65 eV, respectively. Compared to CT‐F, CT‐Cl shows more downshifted HOMO and LUMO levels. Chlorination of CT‐Cl has a more pronounced effect on the LUMO level than on the HOMO level, resulting in a reduced bandgap. This trend is consistent with their optical properties.

### 2.4 Theoretical Calculations

Density functional theory (DFT) calculations were conducted using the B3LYP/6−311G(d,p) basis set to investigate the influence of halogen substitutions on the geometry and energy level distributions. To simplify the calculation, all the alkyl chains are replaced with methyl groups. Both CT‐F and CT‐Cl exhibit similar coplanar structure and molecular electrostatic potential (ESP) distributions, as shown in Figure 2e. Compared to CT‐F, CT‐Cl shows a broader and more negative ESP distribution at the terminal end group due to the chlorine substitution. The HOMO/LUMO energy levels of Y6 are calculated to be −5.87/−3.85 eV, whereas CT‐Cl (−5.77 eV/−3.87 eV) and CT‐F (−5.72 eV/−3.81 eV) show much higher HOMO levels attributed to the stronger electron donating ability of the TT_N_T_N_TT core compared to the D_N_A′_N_D in Y6 (Figure S2, Supporting Information). Chlorine substitution enhances the ICT effect and intermolecular π‐π interactions, ultimately resulting in a narrower band gap for the chlorinated CT‐Cl (Figure S3, Supporting Information).

### 2.5 Single Crystal Analysis

**Figure 3:**
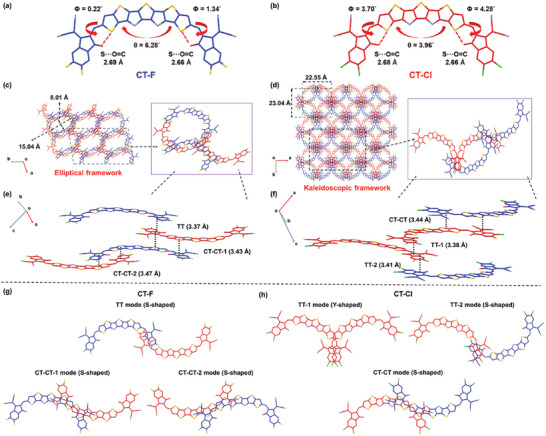
Monomolecular crystallographic structure of a) CT‐F and b) CT‐Cl. 3D interpenetrating packing network of c) CT‐F and d) CT‐Cl observed along the c‐crystallographic axis with insets highlighting the molecular packing in a unit cell; side view of the molecular packing of e) CT‐F and f) CT‐Cl in a unit cell, illustrating the different π–π interaction modes and interlayer π–π stacking distances; eight and three distinct dimeric π–π packing modes observed in the single crystal of g) CT‐F and h) CT‐Cl, respectively. All alkyl chains and H atoms of CT‐F and CT‐Cl are omitted for clarity. Blue and red colors denote different enantiomers in the crystal structures.

The single‐crystal X‐ray diffraction analysis was employed to study the molecular structure and intermolecular packing of the A‐TT_N_T_N_TT‐A‐type NFAs. Single crystals of CT‐F and CT‐Cl were successfully grown using the vapor diffusion method with hexane/chloroform. CT‐F adopts the monoclinic space group P‐1, while CT‐Cl exhibits the triclinic space group C2/c. The crystallographic parameters are detailed in Table S1 (Supporting Information), respectively. Illustrated in **Figure** **3**a,b, similar to A‐D_N_A′_N_D‐A Y6‐based derivatives, CT‐F and CT‐Cl show a distinctive C‐shaped geometry resulting from the conformational locking between the A and TT units in the A‐TT_N_T_N_TT‐A structure through intramolecular non‐covalent interaction involving S and C = O groups.

CT‐F exhibits a smaller dihedral angle of 0.22° between A and D unit with a S···O distance of 2.69 Å, and a larger dihedral angle of 1.34° with a shorter S···O distance of 2.66 Å. Furthermore, CT‐Cl exhibits a larger dihedral angle of 3.70° and 4.28° between A and TT unit at the both sides with S···O distances of 2.68 and 2.66 Å, respectively. Additionally, the torsion angle within the central TT_N_T_N_TT cores is 6.28 and 3.96 degrees for CT‐F and CT‐Cl, respectively.

Both CT‐F and CT‐Cl self‐assemble into a 3D interpenetrating packing network, stacked through multiple π−π interactions (Figure 3c,d). This result reveals that the C‐shaped geometry is essential for maintaining 3D intermolecular packing motifs, regardless of whether the structure is A‐TT_N_T_N_TT‐A or traditional A‐D_N_A′_N_D‐A. The crystal structure of CT‐Cl comprises a unit cell containing four molecules with three distinct dimeric packing configurations (Figure 3f). Depending on the geometry shape of the interacting dimeric complex, a Y‐shaped TT‐1 mode and a S‐shaped TT‐2 mode via terminal‐to‐terminal (TT) π‐π stacking involving Cl^…^Cl and Cl^…^CN intermolecular interactions, and an S‐shaped CT‐CT mode involving core‐to‐terminal (CT) and core‐to‐terminal π‐π stacking are found.

Similarly, CT‐F also features three distinct dimeric packing configurations (Figure 3e), including a S‐shaped TT mode and a pair of S‐shaped CT‐CT1 and CT‐CT2 modes. The corresponding π−π stacking distances in the different modes are indicated in Figure 3e,f.

It should be emphasized that the A‐TT_N_T_N_TT‐A‐type CT‐F and CT‐Cl exhibits have a reduced number of packing modes compared to Y6 derivatives which usually possess at least 4 dimeric packing modes, suggesting improved packing ordering and enhanced crystallinity. When observed from the c‐axis which is approximately perpendicular to the π–π stacking plane, the CT‐F display a 3D lattice structure with elliptical rings of uniform size of 8.01 × 15.04 Å in dimension. (Figure 3c). This indicates that all the overlapping π‐π stacking interactions are superimposed within the 3D framework. In contract, CT‐Cl also shows a distinct circular ring with roughly 22.55 × 23.04 Å in dimension (Figure 3d). However, a circular ring consistently intersects with the other four rings, resulting in the formation of a highly ordered kaleidoscope‐like 3D structure. This demonstrates that altering the end‐group halogen enables the modulation of intermolecular terminal‐to‐terminal and core‐to‐terminal interactions, thus engineering the 3D packing of the CT molecules. The molecular packing coefficient, which quantifies the fraction of the unit cell occupied by molecules, offers insights into the strength of intermolecular interactions and the density of molecular packing. Using Platon software, the packing coefficients for Y6, CT‐F, and CT‐Cl were calculated to be 54.5%, 63.5%, and 65.9%, respectively.^[^ ^17^ ^]^ The higher packing coefficients of CT‐F and CT‐Cl suggest a more compact molecular arrangement, which is advantageous for charge transport. Overall, compared to Y6, CT‐F and CT‐Cl exhibit a more orderly and densely packed structure, driven by stronger intermolecular interactions.

### 2.6 Photovoltaic Performance

**Figure 4:**
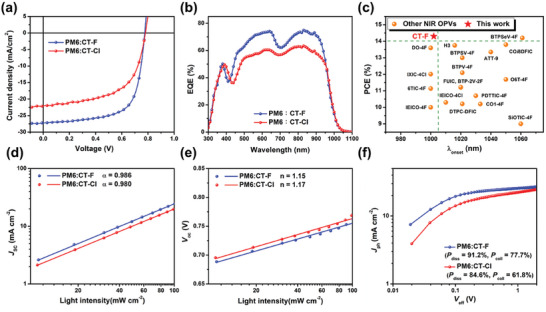
a) *J–V* curves and b) EQE spectra of the PM6:CT‐F and PM6:CT‐Cl‐based devices fabricated using chloroform as the processing solvent; c) Plots of the absorption onset versus PCE in binary OPVs based on previous NIR NFAs (with absorption onset beyond 1000 nm beyond 1000 nm) reported in the literature. d) *J*_sc_ versus light intensity and e) *V*_oc_ versus light intensity of the PM6:CT‐F and PM6:CT‐Cl devices; f) *J*_ph_ versus *V*_eff_ of the optimized devices.

OPV devices with an inverted structure of ITO/ZnO/PM6:NFA/MoO_3_/Ag were fabricated to investigate the photovoltaic performance of CT‐F and CT‐Cl. Active layers were prepared by spin‐coating a 1:1.5 (wt%) solution of PM6:NFA in chloroform. The active layers of the optimized devices were thermally annealed (TA) at 130 °C for 10 min, Their current density–voltage (*J–V*) curves are shown in **Figure** **4**, and corresponding detailed PV parameters are summarized in **Table** **2**. The PM6:CT‐Cl‐based (1:1.5 wt.%) device demonstrated a PCE of 10.05%, with an *V*_oc_ of 0.78 V, a *J*_sc_ of 22.02 mA cm^−2^, and an FF of 58.4%. More significantly, the PM6:CT‐F (1:1.5 wt.%) device achieved a higher PCE of 14.30% with a *V*_oc_ of 0.78 V, a higher *J*_sc_ of 27.21 mA cm^−2^, and a significantly improved FF of 67.6%, showing a broad external quantum efficiency (EQE) response extending from the UV–vis to the NIR regions. The improved performance may be ascribed to the favorable phase separation morphology. The integrated *J*_sc_ values derived from the external quantum efficiency (EQE) measurements closely align with the *J*_sc_ values obtained from *J–V* measurements. This value is the highest reported among the NIR A‐D‐A‐type NFAs used in binary OPVs with an optoelectronic response beyond 1000 nm, as reported in the literature. (Figure 4c and Table S2, Supporting Information). It is anticipated that using a more energy‐aligned P‐type polymer in combination with the CT‐based acceptors could enhance device performance. The NIR properties of CT‐based acceptors hold great promise for tandem and ternary blend OPVs, optimizing UV–vis‐NIR light‐harvesting efficiency and enabling semi‐transparent OPVs for specific applications.

**Table 2:** Photovoltaic parameters of the PM6:NFAs‐based OPVs under AM 1.5G irradiation.

active layer	blend ratio [wt%]	*V*_oc_ [V]	*J*_sc_ [mA cm^−2^]	FF [%]	PCE [%]^a)^
PM6:CT‐F	1:1.5	0.78 (0.78 ± 0.01)	27.21 (25.82 + 1.19)	67.6 (67.8 ± 1.90)	14.30% (13.71 ± 0.31)
PM6:CT‐Cl	1:1.5	0.78 (0.78 ± 0.01)	22.02 (21.15 ± 0.62)	58.4 (57.5 ± 1.26)	10.05% (9.54 ± 0.37)

^a)^ The average PCE values with the standard deviations obtained from 10 individual devices.

### 2.7 Charge Carrier Dynamics Analysis

The parameter α extracted from the *J*_sc_ versus *P*_light_^α^ correlation in Figure 4d, reflects the extent of bimolecular recombination in the OPVs. Higher α values closer to 1 indicate less bimolecular recombination. CT‐F and CT‐Cl devices exhibit α values of 0.986 and 0.980, respectively, suggesting minimal bimolecular recombination in both. Figure 4e depicts the direct correlation between *V*_oc_ and *P*_light_ in the equation *V*_oc_ ∝ n(kT/q)ln(*P*_light_). The slopes of Figure 4e are proportional to 1.15 kT q^−1^ for PM6:CT‐F and 1.17 kT q^−1^ for PM6:CT‐Cl. This suggests less trap‐assisted recombination in the PM6:CT‐F, likely contributing to its greater FF. To gain insight into the effect of halogen substitution on the exciton dissociation and charge collection, the exciton dissociation probability (*P*_diss_) and charge collection efficiency (*P*_coll_) were systemically studied by measuring photocurrent density *J*_ph_ versus effective voltage *V*_eff_ (Figure 4f). The *P*_diss_/*P*_coll_ values are 91.2%/77.7% for PM6: CT‐F and 84.6%/61.8% for PM6: CT‐Cl, respectively. The superior *P*_diss_ and *P*_coll_ of the PM6: CT‐F device indicate more efficient charge dissociation and collection, contributing to the improved *J*_sc_ and FF values.

To investigate the influence of halogen substitution on charge carrier dynamics in the devices, we employed transient photocurrent (TPC) and transient photovoltage (TPV) measurements. (Figure S4, Supporting Information). The PM6:CT‐F device displayed a faster charge extraction time (0.40 µs) compared to the PM6:CT‐Cl device (0.44 µs), signifying improved charge collection efficiency. Moreover, the charge carrier lifetime in PM6:CT‐F (15.6 µs) was longer than in PM6:CT‐Cl device (9.3 µs), indicating reduced charge recombination in CT‐F‐based OPVs. This relates to the higher FF and *J*_sc_ observed in CT‐F devices.

Photoluminescence (PL) quenching experiments were conducted to investigate exciton dissociation and charge separation efficiency in the blends. As shown in Figure S5a (Supporting Information), PM6:CT‐F and PM6:CT‐Cl thin films exhibited near‐complete PL quenching (99.9%) upon excitation of the donor polymer PM6 at 580 nm. This indicates efficient exciton dissociation at the D/A interface in both blends. Furthermore, excitation of the acceptor CT‐F at 875 nm led to a substantial reduction (99.8%) in PL intensity, suggesting efficient transfer of holes from the PM6 donor to the CT‐F acceptor (Figure S5b, Supporting Information). Figure S5c (Supporting Information) also shows efficient PL quenching (i.e., ex. at 900 nm) in the PM6:CT‐Cl blend.

### 2.8 Energy Loss Analysis

To investigate the energy loss (*E*_loss_) mechanisms, Fourier transform photocurrent spectroscopy‐external quantum efficiency (FTPS‐EQE) and electroluminescence (EL) spectra were acquired for PM6:CT‐F‐ and PM6:CT‐Cl‐based OPVs, as shown in Figure S6a,b (Supporting Information), respectively. The detailed energy loss parameters are summarized in Table S3 (Supporting Information). The charge transfer state (*E*_CT_) energies of PM6:CT‐F‐ and PM6:CT‐Cl‐based OPVs, determined by fitting the FTPS‐EQE and EL spectra, were 1.312 and 1.291 eV, respectively. The higher *E*_CT_ in PM6:CT‐F‐based OPVs was attributed to the higher LUMO level of CT‐F. The non‐radiative energy loss (Δ*E*_non‐rad_) was estimated from the external quantum efficiency of electroluminescence (EQE_EL_) using the equation Δ*E*_non‐rad_ = −k_B_ T/q ln (EQE_EL_), as shown in Figure S6c (Supporting Information). The calculated Δ*E*_non‐rad_ values for PM6:CT‐F‐ and PM6:CT‐Cl‐based OPVs were 0.253 and 0.234 eV, respectively. All in all, the *E*_loss_ in PM6:CT‐F‐ and PM6:CT‐Cl‐based OPVs was 0.578 and 0.518 eV, respectively. The slightly lower *E*_loss_ in PM6:CT‐Cl‐based OPVs contributed to the similar *V*_OC_ compared to PM6:CT‐F‐based OPVs.

### 2.9 Contact Angle Measurements

**Figure 5:**
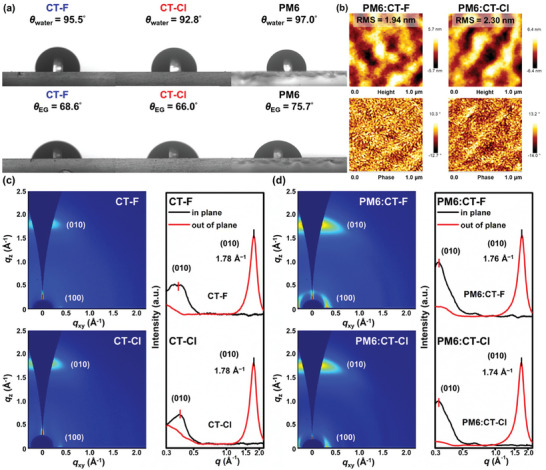
a) Surface contact angle measurements of CT‐F, CT‐Cl, and PM6 neat films using ultra‐pure water and ethylene glycol (EG) as wetting liquids. b) Tapping‐mode atomic force microscopy height images (above) and phase images (below) of PM6:CT‐F and PM6:CT‐Cl thin films. 2D GIWAXS patterns and the corresponding 1D scattering line‐cut profiles along the in‐plane and out‐of‐plane direction of the c) CT‐F and CT‐Cl neat films and the d) PM6:CT‐F and PM6:CT‐Cl blend films thermally annealed at 130 °C for 10 min.

To understand how halogen substitution affects surface energy and miscibility between NFAs and PM6, we measured the contact angles of water and ethylene glycol on pristine films (**Figure** **5**a). The surface energies (𝛾) of PM6, CT‐F, and CT‐Cl were estimated to be 39.1, 48.7, and 61.4 mN m^−1^, respectively, indicating that Cl substitution at the end group significantly enhances the surface energy of CT‐Cl.

According to the Flory–Huggins interaction theory, the interaction parameters (𝜒) derived from (𝛾D^1/2^ – 𝛾A^1/2^)^2^ reflect the miscibility between D and A moieties. The calculated parameter (𝜒) between PM6 and CT‐F (0.53) is significantly lower than that between PM6 and CT‐Cl (2.51). This indicates better miscibility in the PM6:CT‐F blend, leading to a more favorable phase separation morphology. Consequently, the PM6:CT‐F blend exhibited enhanced charge transport compared to the PM6:CT‐Cl blend.

### 2.10 Atomic Force Microscopy

Atomic force microscopy (AFM) was utilized to analyze the surface morphologies of the blend films, as depicted in Figure 5b. The PM6:CT‐F film exhibited a nanofiber‐type surface with a root‐mean‐square (RMS) roughness of 1.94 nm. This suggests the presence of well‐formed, interpenetrating networks that could enhance charge generation and transport, potentially leading to the higher FF and *J*_sc_ observed in the PM6:CT‐F device. Conversely, the PM6:CT‐Cl film displayed a rougher surface with an RMS roughness of 2.30 nm, indicative of more pronounced phase separation.

### 2.11 Thin‐Film Structures Revealed from GIWAXS

Grazing incidence wide‐angle X‐ray scattering (GIWAXS) was employed to investigate the morphology of the neat and blend films. The resulting 2D GIWAXS images and corresponding 1D line‐cut profiles along the in‐plane (*q*_xy_) and out‐of‐plane (*q*_z_) directions are presented in Figure 5c,d and Table S4 (Supporting Information). Both CT‐F and CT‐Cl neat film exhibited strong π–π stacking (010) peaks at *q*_z_ = 1.78 Å^−1^ corresponding to a π–π distance (*d*_π_) of 3.53 Å (Figure 5c). Notably, CT‐Cl displayed enlarged crystalline coherence lengths of π–π stacking (*L*_c‐ππ_ = 19.97 Å) and lamellar stacking (*L*_c‐l_ = 30.15 Å) compared to CT‐F (*L*_c‐ππ_ = 17.28 Å, *L*_c‐l_ = 28.76 Å), which can be attributed to the strengthened intermolecular interactions and crystallinity of CT‐Cl induced by the chlorinated end‐group. The PM6:CT‐F and PM6:CT‐Cl blend films, subjected to thermal annealing at 130 °C for 10 min, (Figure 5d) exhibited face‐on oriented π–π stacking reflections at *q*_z_ = 1.76 and 1.74 Å^−1^corresponding to a *d*_π_ of 3.57 and 3.61 Å, respectively. The slight increase in *d*_π_ spacing in the blend films is likely due to the interpenetration of NFAs within the PM6 matrix. Furthermore, the PM6:CT‐Cl blend film showed a larger *L*_c‐ππ_ value of 20.19 Å than PM6:CT‐F with a *L*_c‐ππ_ value of 19.49 Å, again implying stronger intermolecular interactions of CT‐Cl and thus weakened D‐A interaction. The lower crystallinity of CT‐F compared to CT‐Cl likely facilitates a more favorable intermixed D/A morphology within the PM6:CT‐F blend film, which, in turn, contributes to the enhanced device efficiency. This observation underscores the critical role of end‐group modifications in NFAs for fine‐tuning the balance of intermolecular interactions between NFAs and polymers, ultimately impacting the overall performance of the device.

### 2.12 OPD Characteristics

**Figure 6:**
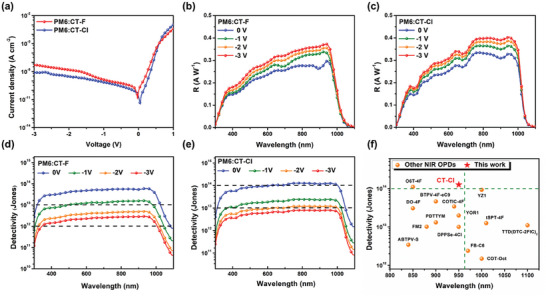
a) Characteristic *J*–*V* curves of PM6:CT‐F and PM6:CT‐Cl OPDs in the dark. Responsivity (R) curves of b) PM6:CT‐F and c) PM6:CT‐Cl‐based devices from 0 to −3 V, respectively. *D*_sh_* curves of d) PM6:CT‐F and e) PM6:CT‐Cl OPDs from 0 to −3 V. f) Plots of absorption versus *D*_sh_* in OPDs based on previous NIR NFAs (absorption onset beyond 1000 nm) reported in the literature.

To evaluate the OPD characteristics of CT‐F and CT‐Cl, we employed the same photodiode structure (ITO/ZnO/PM6:NFAs/MoO_3_/Ag) used for the OPVs. It is important to note that OPD devices typically require a much thicker active layer compared to OPVs to minimize charge injection current. The optimal thickness for the OPD active layer was found to be within the range of 220—250 nm.^[^ ^18^ ^]^ The analysis of dark current‐voltage (*J–V*) curves (shown in Figure S7, Supporting Information and summarized in Table S5, Supporting Information) revealed that a 1:1.2 (PM6:CT‐F/CT‐Cl) blend ratio is optimal for OPDs as well. This weight ratio led to reduced charge injection and improved responsivity across various wavelengths. The dark current (*J*_d_) of the OPD is a critical parameter that influences the level of the detectivity (*D*_sh_*) value. As shown in **Figure** **6**a and **Table** **3**, the PM6:CT‐F‐based (1:1.2 wt.%) OPD exhibited *J*_d_ values of 1.4 × 10^−9^ A cm^−2^ at −1 V and 5.0 × 10^−8^ A cm^−2^ at −3 V reverse bias. The PM6:CT‐Cl‐based (1:1.2 wt.%) device demonstrated the notably lower *J*_d_ values of 2.1×10^−11^, 7.1 × 10^−10^, and 7.8 × 10^−9^ A cm^−2^ under reverse bias at 0, −1, and −3 V, respectively. Compared to CT‐F, which has a lower surface energy, CT‐Cl with its higher surface energy tends to accumulate on the bottom side of the PM6:CT‐Cl active layer, leading to more pronounced vertical phase segregation in thick films. As illustrated in Figure S8 (Supporting Information), secondary ion mass spectrometry (SIMS) was used to investigate vertical composition by analyzing the distribution of cyano (CN) functional groups in the two material blends. The results confirmed that the CN moieties in the CT‐Cl blend are located deeper within the film compared to those in the CT‐F blend. This leads to an enrichment of CT‐Cl near the ITO/ZnO interface (cathode) and a higher concentration of the PM6 polymer near the MoO_3_/Ag interface (anode). Such a vertical distribution increases charge injection barriers and effectively blocks the unfavorable reverse hole and electron carrier injection.^[^ ^19^ ^]^ Another factor is the deeper‐lying HOMO level of CT‐Cl (−5.60 eV) compared to that of CT‐F (−5.56 eV), which effectively suppresses hole injection from the cathode under reverse bias, thereby reducing the dark current.^[^ ^19^, ^20^ ^]^ The notably lower *J*_d_ values observed in the PM6:CT‐Cl‐based OPD device may be also related to the *E*_CT_ and the distribution of bandtail states. Ng and co‐workers have suggested that the *E*_CT_ and the distribution of bandtail states are the dominant factors affecting noise and dark current.^[^ ^21^ ^]^ The wide distribution of bandtail states, which is attributed to the serious energy disorder and non‐radiative energy loss in the device, can be reflected by the Urbach energy (*E*_U_).^[^ ^9c^ ^]^ The *E*_U_ values of PM6:CT‐Cl‐ and PM6:CT‐F‐based devices, obtained by fitting the low‐energy tail of the FTPS‐EQE spectrum, are 53.9 and 55.5 meV, respectively. The higher *E*_CT_ in the PM6:CT‐F‐based device provides a potential energy barrier for thermal carrier generation, while the larger Δ*E*_non‐rad_ and *E*_U_ values indicate more severe bandtailing. In contrast, the stronger molecular packing of CT‐Cl suppresses Δ*E*_non‐rad_ and *E*_U_, resulting in a lower *J*_d_ obtained in PM6:CT‐Cl‐based OPD.

**Table 3:** Photoresponse parameters (*J*_d_, R, and *D*_sh_*) of the OPDs under different biases.

Device	bias [V]	*J*_d_ [A cm^−2^]	R [A W^−1^]^a)^	*D*_sh_* [Jones]^a)^
PM6:CT‐F	0	7.45 × 10^−11^	0.29	6.0 × 10^13^
	−1	1.4 × 10^−9^	0.33	1.6 × 10^13^
	−3	5.0 × 10^−8^	0.37	2.9 × 10^12^
PM6:CT‐Cl	0	2.1 × 10^−11^	0.33	1.3 × 10^14^
	−1	7.1 × 10^−10^	0.37	2.4 × 10^13^
	−3	7.8 × 10^−9^	0.40	8.1 × 10^12^

^a)^ R and *D*_sh_* at 950 nm.

The responsivity (*R*) is expressed as the ratio of the photocurrent to the incident light intensity, which can be calculated as follows,

(1) $$R\;\left(\lambda \right)=\;\mathrm{EQE}\;\left(\lambda \right)\times \frac{q\lambda }{hc}\;\left(AW^{-1}\right)$$

where λ is the wavelength of incident light, *q* is the elementary charge, h is the Planck constant, and c is the speed of light.^[^ ^22^ ^]^

Figure 6b,c demonstrate the *R* curves of PM6:CT‐F and PM6:CT‐Cl‐based OPDs under different bias. The PM6:CT‐F‐based OPD exhibited *R* of 0.33 and 0.37 A W^−1^ at 950 nm under −1 and −3 V bias, respectively. In comparison, the PM6:CT‐Cl‐based OPD displayed a higher R of 0.37 and 0.40 A W^−1^ at 950 nm under −1 and −3 V bias, respectively. The CT‐Cl OPD showed a red‐shifted onset wavelength (1042 nm) compared to CT‐F (1034 nm) device due to the chlorinated end group.

The shot‐noise‐limited specific detectivity (*D*_sh_*) quantifies a detector's sensitivity to weak optical signals and is calculated using the following equation:

(2) $${D_{\mathrm{sh}}}^{*}=\frac{R}{\sqrt{2q\;J_{d}}}$$

where R is the responsivity, *q* is the elementary charge, and *J*_d_ is the dark current density.^[^ ^13c^ ^]^ As shown in Figure 6d,e, the PM6:CT‐F‐based OPD exhibited a *D*_sh_* value over 10^12^ jones with a peak of 1.6 × 10^13^/2.9 × 10^12^ Jones at 950 nm under a reverse bias of −1/−3 V. Consistently, the PM6:CT‐Cl‐based OPD exhibited ultra‐high *D*_sh_* values, surpassing 10^14^ Jones in the NIR region from 620 to 1000 nm under 0 V with a peak value of 1.3 × 10^14^ Jones at 950 nm. Under a reverse bias of −1 V, the PM6:CT‐Cl‐based OPD maintained high *D*_sh_* values exceeding 10^13^ jones from 360 to 1020 nm, with a peak of 2.4 × 10^13^ Jones at 950 nm. Enhancing the reverse bias to −3 V can also maintain the *D*_sh_* values exceeding 10^12^ jones from 320 to 1040 nm. (Table 3).

The PM6:CT‐F and PM6:CT‐Cl‐based OPDs exhibit high *D*_sh_* values based on shot noise. Furthermore, we also measured the white noise (*i*_n_) to determine the specific detectivity *D*_n_* values more accurately, which are calculated using the following equation: Therefore, we measure the white noise (*i*_n_) to determine the *D*_n_* values more accurately, which are calculated using the following equation:

(3) $${D_{n}}^{*}=\frac{R\sqrt{A\Delta f}}{i_{n}}$$

where R is the responsivity, A is the active area of photodetector, ∆f is the electrical bandwidth, and *i*_n_ is the white noise current.^[^ ^23^ ^]^ The actual noise of the OPD, including thermal, Johnson, and 1/f noise, was measured at an applied bias of −3 V and is presented in Figure S9a (Supporting Information). The *i*_n_ was calculated as the root‐mean‐square value of the noise current within the frequency range of 100 to 1000 Hz. The *i*_n_ of PM6:CT‐F‐ and PM6:CT‐Cl‐based OPDs were 1.5 × 10^−12^ and 2.2 × 10^−13^ A Hz^−1/2^, respectively. By employing white noise for calculation,^[^ ^24^ ^]^ the *D*_n_* of PM6:CT‐F‐ and PM6:CT‐Cl‐based OPDs were determined, as shown in Figure S9b (Supporting Information). The maximum *D*_n_* values achieved for PM6:CT‐F‐ and PM6:CT‐Cl‐based OPDs were 7.8×10^10^ and 5.8×10^11^ Jones, respectively.

**Figure 7:**
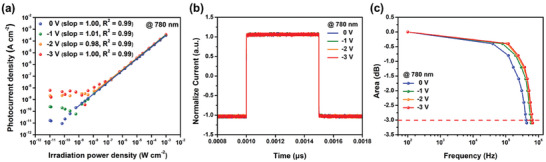
a) LDR curves, b) the rise/fall times, c) cut‐off frequencies (*f*_−3dB_) of the PM6:CT‐Cl‐based OPD using a 780 nm light source.

The linear dynamic range (LDR) of the CT‐Cl‐based OPD was measured under illumination from 520 (Figure S10a, Supporting Information) and 780 nm (**Figure** **7**a) light sources at varying light intensities. The LDR values are calculated using the following equation:

(4) $$\mathrm{LDR}\;\left(\mathrm{dB}\right)=20\times \log\left(\frac{J_{max}}{J_{min}}\right)$$

where *J*_max_ and *J*_min_ are the current densities under the highest and lowest light intensities, respectively.^[^ ^25^ ^]^ The measured LDR values for the CT‐Cl OPD under 780 nm illumination were 124.0, 115.9, 98.8, and 83.8 dB at biases of 0, −1, −2, and −3 V, respectively, as summarized in Table S6 (Supporting Information). The high LDR values indicate that the device is effective at detecting low‐intensity light, making it promising for scotopic vision applications. The PM6:CT‐Cl‐based OPD demonstrated excellent response characteristics under both 520 and 780 nm light sources. A digital oscilloscope (Teledyne LeCroy, WaveRunner 625Zi) was employed to record the pulsed optical signals detected by the OPDs. LED lamps emitting light (520 and 780 nm) with an irradiance of 1 mW cm^−2^ were utilized as the incident light sources at biases of 0, −1, −2, and −3 V. The rise and fall response times of the OPDs were extracted from Figure 7b (780 nm) and Figure S10b (Supporting Information) (520 nm), respectively. These times were determined by measuring the duration required for the output optical signal to increase from 10% to 90% of its peak value (rise time) and decrease from 90 to 10% of its peak value (fall time). Under 780 nm illumination, the rise and fall times of the PM6:CT‐Cl‐based OPDs were determined to be 0.35/0.21, 0.33/0.11, 0.30/0.11, and 0.29/0.10 µs at biases of 0, −1, −2, and −3 V, respectively. The values are comparable to those of commercially available silicon photodiodes (S1336‐44BQ) which meets the requirement of commercial application (i.e., video recording, imaging applications, and specific applications requiring a NIR response).^[^ ^18^ ^]^ The cut‐off frequency (*f*_−3dB_) is defined as the frequency at which the amplitude of the output signal of the photo‐response is −3 dB, as illustrated in Figure 7c (780 nm) and Figure S10c (Supporting Information) (520 nm). When utilizing a 780 nm light source, the *f*_−3dB_ values are found to be 420, 590, 630, and 650 kHz at biases of 0, −1, −2, and −3 V respectively. It should be noted that the *f*_−3dB_ values for solution‐processed OPDs typically fall within the 10−100 kHz range. To validate the *f*_–3dB_ values, we measure *f*_RC_, which is determined by the RC time constant and found to be comparable to *f*_–3 dB_. The transit time limited cut‐off frequency (*f*_tr_) could be neglected in this case. The relationship between RC limited cut‐off frequency (*f*_RC_) and the RC time constant can be expressed as:^[^ ^26^ ^]^

(5) $$f_{RC,-3dB}=\frac{1}{2\pi R_{s}C}$$

where R_s_ and C are OPD's total series resistance and capacitance, respectively. The value of Rs and C are extracted from electrochemical impedance spectroscopy (EIS) measurements, as shown in Figure S11 (Supporting Information). The *f*_RC_ of PM6:CT‐Cl‐based OPDs at 0, −1, −2, −3 V were 635.5, 757.4, 780.4, and 804.3 kHz, respectively, which is comparable to *f*_–3dB_ values. The values obtained in this study exceed several hundred kHz across a broad range of wavelengths,^[^ ^2b^ ^]^ comparable to those of commercial Si‐PD (S1336‐44B (0.5 MHz)).^[^ ^25^ ^]^ The CT‐Cl‐based OPD surpasses most of the high‐performance NIR OPDs reported in the literature, as summarized in Figure 6f and Table S7 (Supporting Information), particularly in terms of *D*_sh_* and rise/fall times.

## 3 Conclusions

We have designed and synthesized a highly electron‐rich S,N heptacyclic TT_N_T_N_TT heteroacene through palladium‐catalyzed Negishi coupling and Buchwald‐Hartwig amination. The highly electron‐donating TT_N_T_N_TT building block was coupled with FIC and Cl‐IC acceptors to furnish two new NIR electron acceptors CT‐F (*E*_g_^opt^ = 1.24 eV) and CT‐Cl (*E*_g_^opt^ = 1.22 eV) exhibiting absorption beyond 1000 nm as a result of the effective ICT effect. Stacked through multiple π−π interactions in the single crystal structures, C‐shaped CT‐F self‐assembles into a 3D intermolecular packing network with elliptical rings of uniform size, while CT‐Cl forms a highly ordered kaleidoscope‐like 3D structure with improved packing order and enhanced crystallinity. When combined with the wide bandgap polymer PM6, the CT‐F‐based binary OPV device achieved an impressive PCE of 14.30% due to the formation of a favorable D‐A intermixed morphology. This performance ranks among the best for NIR OPVs employing A‐D‐A‐type acceptors with photoresponses exceeding 1000 nm. The exceptional NIR responsivity makes CT‐based materials promising for NIR‐OPDs. CT‐Cl possesses a higher surface energy than CT‐F, promoting vertical phase segregation and resulting in its preferential accumulation near the bottom interface of the blend. This arrangement, CT‐Cl‐rich cathode and PM6‐rich anode, combined with the lower‐lying HOMO energy level of CT‐Cl, effectively blocks the undesired hole and electron injection under reverse voltage. Consequently, the PM6:CT‐Cl‐based OPD device achieved a high responsivity of 0.40 AW^−1^ at 950 nm at −3 V and exhibited ultra‐high *D*_sh_* values exceeding 10^14^ Jones in the NIR region from 620 to 1000 nm, reaching an unprecedentedly high value of 1.3×10^14^ Jones at 950 nm. When utilizing a 780 nm light source, the PM6:CT‐Cl‐based OPDs showed record‐high rise and fall times of 0.33/0.11 µs and an exceptional cut‐off frequency (*f*_‐3dB_) of 590 kHz at −1 V. These results demonstrate that our device exhibits comparable or even superior performance metrics (such as *J*_d_, detectivity, and rise/fall time) when compared to high‐performance organic‐based NIR photodiode‐type OPDs reported in the literature, providing a competitive alternative to commercial silicon photodiodes. The S,N heteroacene has been demonstrated as a superior building block for designing NIR NFAs, paving the way for promising applications in tandem and ternary solar cells, semi‐transparent OPVs, as well as bioimaging, which are currently ongoing in our laboratory.

## Conflict of Interest

The authors declare no conflict of interest.

## Supporting information



Supporting Information

## Data Availability

The data that support the findings of this study are available from the corresponding author upon reasonable request.
