# Supplementary material for: Electron‐Rich Heptacyclic S,N Heteroacene Enabling C‐Shaped A‐D‐A‐type Electron Acceptors With Photoelectric Response beyond 1000 Nm for Highly Sensitive Near‐Infrared Photodetectors
Source: Adv Sci (Weinh). 2025 Jan 14;12(9):2413045. doi: 10.1002/advs.202413045 (PMC11884573; doi:10.1002/advs.202413045)
Supplement: Supplementary file 1 — Supporting Information [file ADVS-12-2413045-s001.docx]

**Electron-Rich Heptacyclic S,N heteroacene enabling C-Shaped A-D-A-type Electron Acceptors with Photoelectric Response Beyond 1000 nm for Highly Sensitive Near-Infrared Photodetectors**

Kuo-Hsiu Huang,^1^ Bing-Huang Jiang,^2^ Han-Cheng Lu,^1^ Yung-Jing Xue,^1^ Chia-Fang Lu,^1^ Yung-Yung Chang,^1^ Ching-Li Huang,^1^ Su-Ying Chien,^3^ Chih-Ping Chen,*^2,4^ and Yen-Ju Cheng*^1,5^

1 K-H Huang, H-C Lu, Dr. Y-J Xue, C-F Lu, Y-Y Chang, Dr. C-L Huang, Prof. Y-J Cheng
Department of Applied Chemistry
National Yang Ming Chiao Tung University
Hsinchu 30010, Taiwan
E-mail: yjcheng@nycu.edu.tw

2 Dr. B-H Jiang, Prof. C-P Chen
Department of Materials Engineering
Ming Chi University of Technology
New Taipei City 243303, Taiwan
E-mail: cpchen@mail.mcut.edu.tw

3 Dr. S-Y Chien
Instrumentation Center
National Taiwan University
Taipei 10617, Taiwan

4 Prof. C-P Chen
College of Engineering and Center for Sustainability and Energy Technologies
Chang Gung University
Taoyuan City 33302, Taiwan

5 Prof. Y-J Cheng
Center for Emergent Functional Matter Science
National Yang Ming Chiao Tung University
Hsinchu 30010, Taiwan

K.-H. Huang and B.-H. Jiang contributed equally to this work.

**Table of contents**

[1. Materials, instruments and characterization S2](#_Toc177668607)

[2. Synthetic procedures S3](#_Toc177668608)

[3. Cyclic voltammetry (CV) characteristics S6](#_Toc177668609)

[4. Density functional theory (DFT) calculation of frontier molecular orbitals S6](#_Toc177668610)

[5. Single crystal growth and the crystallographic data S7](#_Toc177668611)

[6. Photovoltaic device fabrication and characterization S10](#_Toc177668612)

[10. The photoluminescence (PL) quenching characteristics S13](#_Toc177668613)

[11. Fabrication and characterization of OPD devices S14](#_Toc177668614)

[12. ^1^H and ^13^C NMR spectra S19](#_Toc177668615)

[13. References S29](#_Toc177668616)

# Materials, instruments and characterization

All reagents and chemicals were purchased from commercial sources and were used without further purification unless noted otherwise. PM6 was purchased from Solarmer, Inc. Tetrahydrofuran (THF) was dried by solvent purification system. *N*, *N*-dimethylformamide (DMF) was dried by activated molecular sieves. **TT-2Br** was prepared according to the literature procedures.^[1] 1^H and ^13^C NMR spectra were measured using Varian-400 MHz and JEOL-400 MHz instrument spectrometer. Deuterated chloroform (CDCl_3_) with TMS as internal and benzene (C_6_D_6_) were used as references unless otherwise stated. Chemical shifts (*δ*) are reported in parts per million. The mass spectra of the samples were recorded on JEOL T200-GC high resolution spectrometer using field desorption (FD) method. UV-vis absorption spectra were measured on HP8453 UV-vis spectrophotometer. The neat films were prepared by spin-coating chloroform solution of the materials (10 mg mL^-1^) at 1000 rpm for 30 s on the quartz substrate. Differential scanning calorimetry (DSC) and thermogravimetric analysis (TGA) were conducted on a TA DSC250 Instrument and a TA TGA55 Instrument under nitrogen atmosphere at heating/cooling rate of 10 °C/min. Surface topography was investigated using AFM (Bruker Dimension Edge) and standard tips (Tapping mode; Length: 150 μm; Resonance Frequency: 160 kHz; Force Constant: 7.4 N/m).

# Synthetic procedures


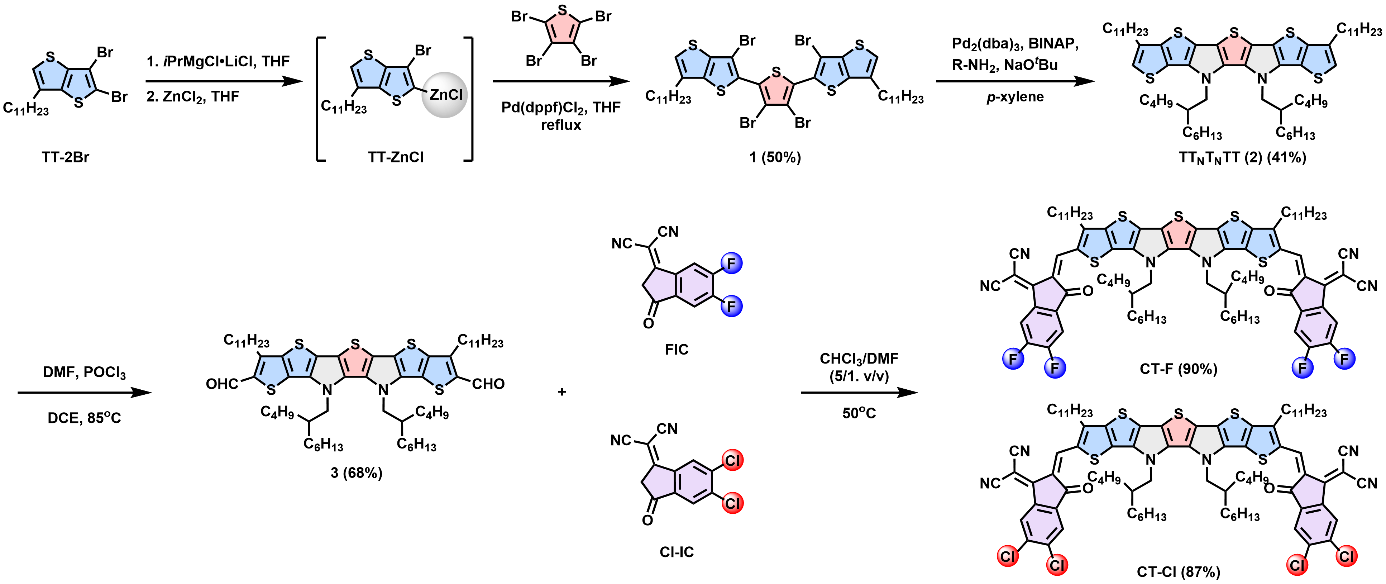


**Synthesis of compound 1**

**
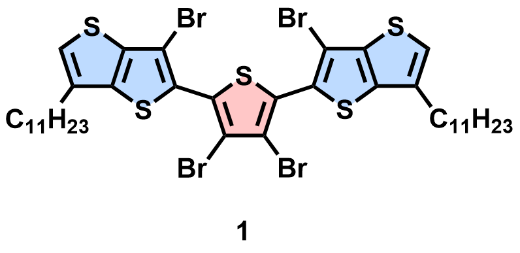
**

To a solution of **TT-2Br** (452 mg, 1.00 mmol) in dry THF (2.0 mL) under nitrogen was added isopropylmagnesium chloride lithium chloride complex solution (1.3 M in THF, 0.85 mL, 1.10 mmol) dropwise at 0 ^o^C. After stirring at 0 ^o^C for 1 h, the solution of zinc chloride (150 mg, 1.10 mmol) in dry THF (2.2 mL) was added to the mixture at 0 ^o^C and stirred for 1 h to form the **TT-ZnCl** intermediate. To a solution of tetrabromothiophene (160 mg, 0.40 mmol) and Pd(dppf)Cl_2_ (29 mg, 0.04 mmol) in dry THF (2.0 mL) was added the freshly prepared **TT-ZnCl** solution slowly at 0 ^o^C. The mixture was refluxed for 16 h. After cooling to room temperature, the reaction solution was quenched by water and extracted with ethyl acetate (25 mL x 2) and water (25 mL). The collection organic layer was dried over anhydrous MgSO_4_. After removing the solvent by reduced pressure, the residue was purified by column chromatography on silica gel (hexane) to get a yellow solid **1** (197 mg, 50%). ^1^H NMR (400 MHz, CDCl_3_): *δ* 7.11 (s, 2 H), 2.73 (t, *J* = 7.6 Hz, 4 H), 1.81–1.69 (m, 4 H), 1.44–1.20 (m, 32 H), 0.88 (m, 6 H). ^13^C NMR (100 MHz, CDCl_3_): *δ* 140.78, 139.18, 136.06, 132.03, 128.35, 123.48, 116.83, 106.07, 32.06, 29.88, 29.79, 29.77, 29.69, 29.50, 29.49, 29.45, 28.79, 22.84, 14.28; HMRS (FD, C_38_H_48_S_5_Br_4_): calcd, 979.9099; found 979.9108.

**Synthesis of compound TT_N_T_N_TT (2)**


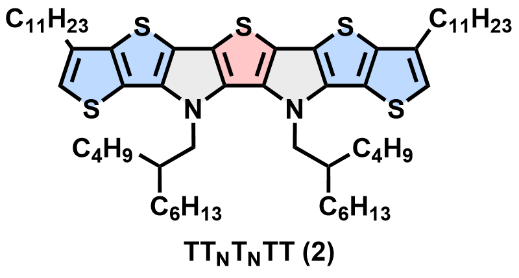


To a mixture of compound **1** (197 mg, 0.2 mmol), sodium *tert*-butoxide (173 mg, 1.8 mmol), BINAP (99.6 mg, 0.16 mmol), and Pd_2_(dba)_3_ (36.6 mg, 0.04 mmol) in degassed *p*-xylene (4.0 mL) was added 2-butyl octan-1-amine (222 mg, 1.2 mmol). The mixture was stirred for 20 h at 110 ℃. The reaction solution was filtered through celite and extracted with ethyl acetate (25 mL x 2) and water (50 mL). The collection organic layer was dried over anhydrous MgSO_4_. After removing the solvent by reduced pressure, the residue was purified by column chromatography on silica gel (hexane) to get a yellow solid **TT_N_T_N_TT (2)** (84.6 mg, 41%). ^1^H NMR (400 MHz, C_6_D_6_): *δ* 6.58 (s, 2 H), 4.55 (d, *J* = 4.7 Hz, 4 H), 2.57 (t, *J* = 7.5 Hz, 4 H), 2.30–2.42 (m, 2H), 1.75–1.64 (m, 4 H), 1.45–1.20 (m, 42 H), 1.20–1.00 (m, 20 H), 1.00–0.87 (m, 8 H), 0.87–0.72 (m, 13 H). ^13^C NMR (100 MHz, C_6_D_6_): *δ* 139.28, 136.98, 136.76, 130.34, 124.10, 119.16, 118.38, 117.90, 54.92, 39.96, 32.39, 32.03, 32.01, 31.68, 31.64, 31.61, 32.39, 32.03, 32.01, 31.68, 31.64, 31.61, 30.25, 30.23, 30.17, 30.15, 30.06, 29.98, 29.94, 29.91, 29.87, 29.75, 29.10, 28.92, 28.87, 26.66, 26.60, 23.39, 23.35, 23.17, 23.04, 23.03, 14.43, 14.34, 14.09, 14.07; HMRS (FD, C_62_H_98_N_2_S_5_): calcd, 1030.6339； found, 1030.6343.

**Synthesis of compound 3**


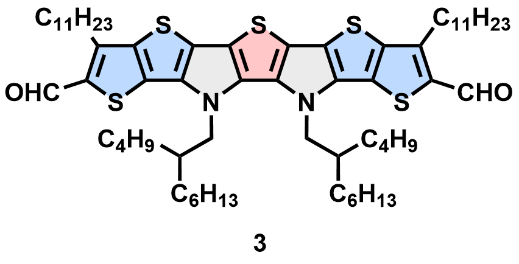


POCl_3_ (76.7 μL, 0.82 mmol) was added to DMF (1 mL) and stirred for 30 min at 0 ℃. To a solution of compound **TT_N_T_N_TT (2)** (84.6 mg, 0.08 mmol) in dichloroethane (8.2 mL) was added the mixture dropwise and stirred for 16 h at 85 ℃. The reaction was poured into water and stirred for 30 min. The reaction solution was extracted with dichloromethane (25 mL x 2) and water (50 mL). The collection organic layer was dried over anhydrous MgSO_4_. After removing the solvent by reduced pressure, the residue was purified by column chromatography on silica gel (hexane/DCM, v/v, 1/1) to get sticky fluorescent orange solid **3** (60.7 mg, 68%). ^1^H NMR (400 MHz, CDCl_3_): *δ* 10.06 (s, 2 H), 4.40 (d, *J* = 8.0 Hz, 4 H), 3.11 (t, *J* = 7.6 Hz, 4 H), 2.15–2.04 (m, 2 H), 1.90–1.81 (m, 4 H), 1.45–1.00 (m, 64 H), 0.91–0.84 (m, 6 H), 0.80–0.70 (m, 12 H). ^13^C NMR (100 MHz, CDCl_3_): *δ* 181.48, 146.88, 140.05, 136.24, 135.72, 131.70, 130.00, 123.12, 119.63, 54.68, 39.77, 32.04, 31.64, 31.63, 31.16, 31.12, 31.06, 31.01, 30.47, 29.76, 29.72, 29.65, 29.62, 29.59, 29.49, 29.45, 28.50, 28.47, 28.11, 26.23, 26.20, 23.05, 23.03, 22.82, 22.66, 14.26, 14.13, 13.90, 13.89; HMRS (FD, C_64_H_98_N_2_O_2_S_5_): calcd, 1086.6237；found 1086.6238.

**Synthesis of CT-F**

**
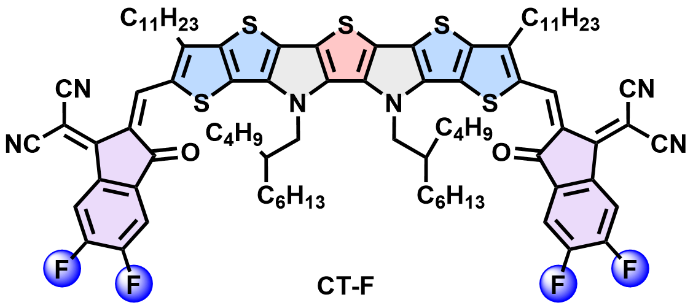
**

To a mixture of compound **3** (60.7 mg, 0.06 mmol) and 2-(5,6-difluoro-3-oxo-2,3-dihydro-1*H*-inded-1-ylidene)malononitrile (51.3 mg, 0.22 mmol) in chloroform/DMF (6.7 ml, 5/1, v/v) was added trimethylsilyl chloride (1.42 ml, 11 mmol) dropwise. The mixture was stirred at 50 ℃ for 16 h. The reaction solution was extracted with dichloromethane (50 mL x 2) and water (75 mL). The collection organic layer was dried over anhydrous MgSO_4_. After removing the solvent by reduced pressure, the residue was purified by column chromatography on silica gel (hexane/DCM, v/v, 1/1) to get a dark blue solid **CT-F** (75.9 mg, 90%). ^1^H NMR (400 MHz, CDCl_3_): *δ* 8.93 (s, 2 H), 8.43 (s, 2 H), 7.63 (s, 2 H), 4.51 (d, *J* = 3.6 Hz, 4 H), 3.11 (t, *J* = 7.2 Hz, 4 H), 2.26–2.12 (m, 2 H), 1.86–1.71 (m, 4 H), 1.52–1.02 (m, 64 H), 0.92–0.73 (m, 18 H). ^13^C NMR (100 MHz, CDCl_3_): *δ* 186.17, 158.45, 155.48, 153.72, 152.98, 142.97, 137.66, 136.62, 136.34, 134.45, 134.30, 133.72, 132.77, 127.00, 122.54, 118.70, 115.33, 114.95, 114.73, 112.25, 112.07, 67.36, 54.99, 40.04, 32.06, 31.83, 31.47, 31.33, 31.14, 29.89, 29.80, 29.77, 29.63, 29.58, 29.49, 28.98, 28.92, 26.70, 26.64, 23.26, 22.84, 22.74, 14.27, 14.22, 14.09; HMRS (FD, C_88_H_102_N_6_O_2_F_4_S_5_): calcd, 1510.6609; found 1510.6605.

**Synthesis of CT-Cl**

**
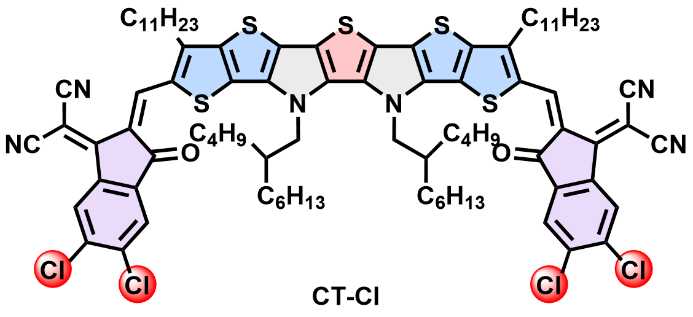
**

To a mixture of compound **3** (60.7 mg, 0.06 mmol) and 2-(5,6-dichloro-3-oxo-2,3-dihydro-1*H*-inded-1-ylidene)malononitrile (51.3 mg, 0.22 mmol) in chloroform/DMF (6.7 ml, 5/1, v/v) was added trimethylsilyl chloride (1.42 ml, 11 mmol) dropwise. The mixture was stirred at 50 ℃ for 16 h. The reaction solution was extracted with dichloromethane (50 mL x 2) and water (75 mL). The collection organic layer was dried over anhydrous MgSO_4_. After removing the solvent by reduced pressure, the residue was purified by column chromatography on silica gel (hexane/DCM, v/v, 1/1) to get a dark blue solid **CT-Cl** (79.2 mg, 87%). ^1^H NMR (400 MHz, CDCl_3_): *δ* 8.85 (s, 2 H), 8.59 (s, 2 H), 7.86 (s, 2 H), 4.50 (d, *J* = 8.1 Hz, 4 H), 3.09 (t, *J* = 7.7 Hz, 4 H), 2.23 (m, 2 H), 1.82–1.72 (m, 4 H), 1.49–1.10 (m, 64 H), 0.90–0.78 (m, 18 H). ^13^C NMR (100 MHz, CDCl_3_): *δ* 186.15, 158.18, 153.81, 143.06, 139.08, 138.79, 137.56, 136.47, 136.11, 134.58, 133.85, 133.14, 127.20, 126.68, 124.65, 122.63, 118.66, 115.39, 115.01, 67.39, 54.93, 39.92, 32.07, 31.91, 31.57, 31.36, 31.09, 29.93, 29.89, 29.79, 29.77, 29.64, 29.57, 29.49, 29.04, 26.81, 23.27, 22.85, 22.77, 14.28, 14.13, 0.15; HMRS (FD, C_88_H_102_N_6_O_2_F_4_S_5_): calcd, 1574.5427；found 1574.5430.

# Cyclic voltammetry (CV) characteristics

CV was conducted on a CH instruments electrochemical analyzer. A carbon glass was used as the working electrode and an Ag/Ag^+^ electrode as the reference electrode, while 0.1 M tetrabutylammonium hexafluorophosphate in acetonitrile was the electrolyte. CV curves were calibrated using ferrocene as the standard, whose HOMO energy level is set at −4.8 eV with respect to zero vacuum level. The HOMO energy levels were obtained from the equation *E*_HOMO_ = −| *E*onset ox − *E*onset ferrocene + 4.8| eV. The LUMO energy levels were obtained from the equation *E*_LUMO_ = −| *E*onset red − *E*onset ferrocene + 4.8| eV.


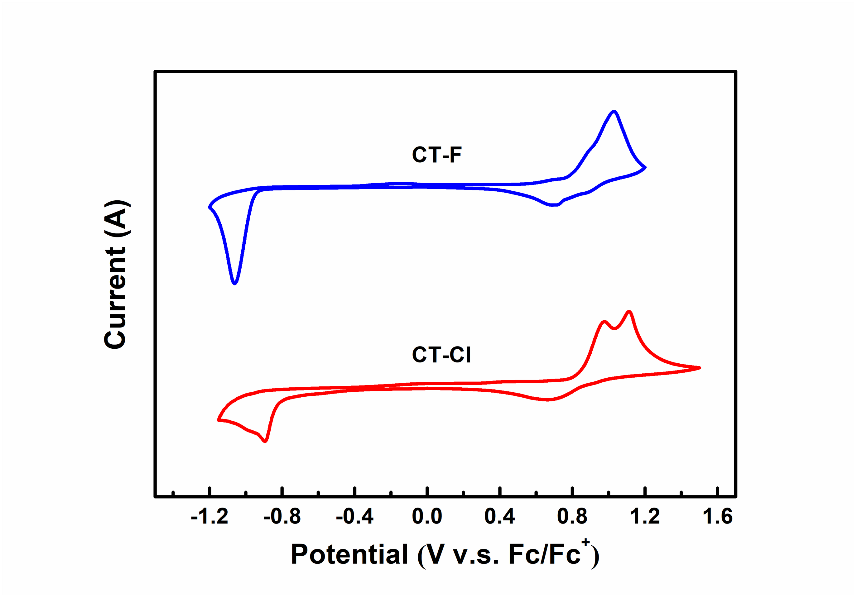


**Figure S1.** Cyclic voltammogram of CT-F and CT-Cl thin films at a scan rate of 100 mV s^-1^.

# Density functional theory (DFT) calculation of frontier molecular orbitals

Computational model compounds for CT-F, CT-Cl and Y6 in which all alkyl chains are simplified into methyl groups, were calculated using Gaussian09 suite15 at the B3LYP/6-311G(d,p) level.


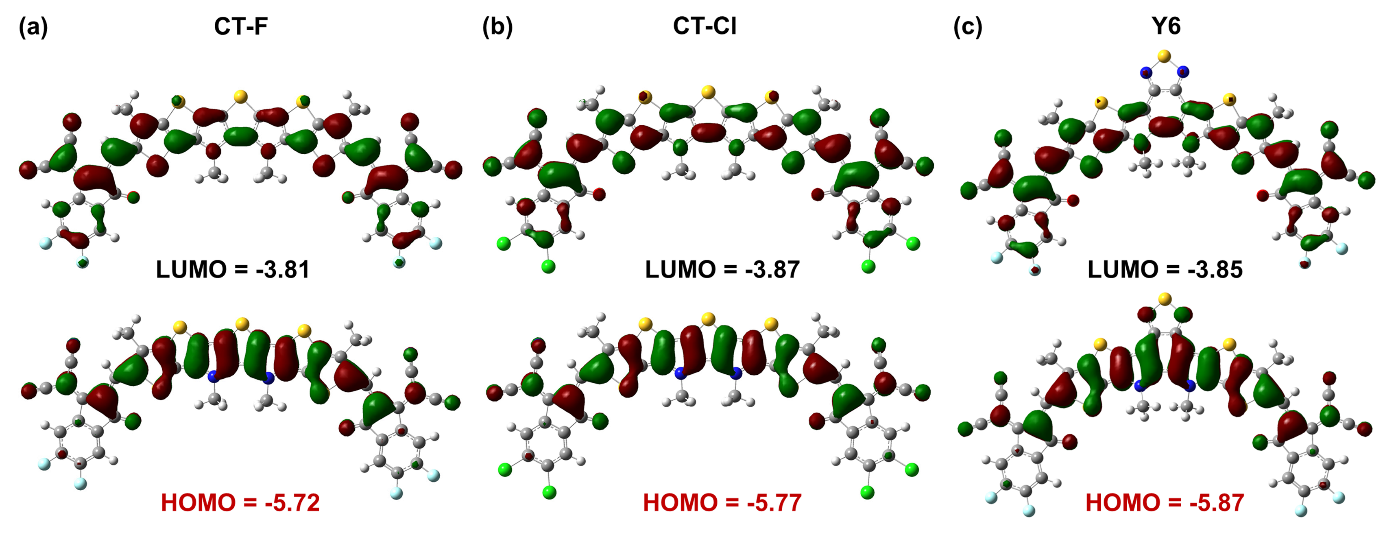


**Figure S2.** Frontier molecular orbital plots of CT-F, CT-Cl and Y6 calculated by DFT at B3LYP/6-311G(d,p).


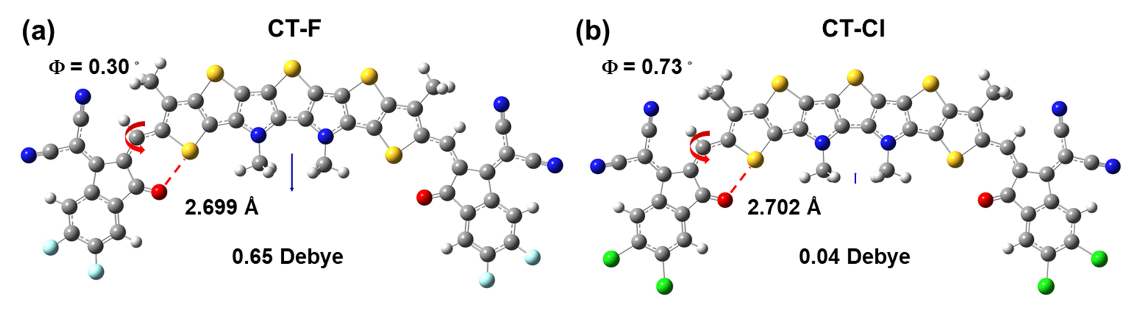


**Figure S3.** Top view of the optimized geometry of CT-F and CT-Cl computed with B3LYP/6-311G(d,p). The intramolecular S–O distances, the D-A dihedral angles (Φ) and dipole moments are calculated. The atomic species C labeled by gray, N by blue, O by red, F by green and S by yellow.

# Single crystal growth and the crystallographic data

**5.1 Single crystal growth**

Two solutions prepared from 1.6 mg of CT-F in 1.0 mL chloroform as well as from 1.3 mg of CT-Cl in 1.6 mL chloroform were separately transferred into a 4 mL vial, which was capped with a piece of an aluminum foil with a hole punched using a 18G needle in the middle. Following that, the vial was placed in a 20 mL vial containing 2.0 mL hexane for CT-F and a 20 mL vial containing 2.5 mL hexane for CT-Cl, respectively. Both vials were sealed tightly, and left standing about 3~5 days for both CT-F and CT-Cl to give small crystal clusters in each vial.

**5.2 Crystallographic data**

Two black single crystals were mounted on a CryoLoop with Parabar 10312 oil for the single-crystal X-ray diffraction experiments at -73 ℃. The crystal size is 0.284 × 0.124 × 0.059 mm^3^ for CT-F and 0.535 × 0.191 × 0.138 mm^3^ for CT-Cl. The single-crystal X-ray diffraction data of CT-F and CT-Cl were individually collected in-house on a Bruker D8 Venture diffractometer equipped with a Mo-target (K_α_ = 0.71073 Å) microfocus X-ray generators and a PHOTON-II CMOS detector. The temperature was adjusted with a nitrogen flow (Oxford Cryosystems, 800+ series). After collection, the cell refinement and the data integration were carried out by Bruker SAINT^[2]^ software package using a narrow-frame algorithm and were corrected for absorption effects using the Multi-Scan method (SADABS).^[3]^ Moreover, the molecular structure was solved by SHELXT (Sheldrick 2015) and refined by SHELXL-2019/1 (Sheldrick, 2019).^[4]^ The final anisotropic full-matrix least-squares method was used to refine on F^2^ with variables parameters to determine crystal structure. All calculations were performed using the APEX4^[5]^ software package. The crystallographic data of CT-F and CT-Cl are listed in Table S1.

**Table S1.** The crystallographic data of CT-F and CT-Cl.

| Identification code | CT-F | CT-Cl |
| --- | --- | --- |
| Empirical formula | C_88_H_102_F_4_N_6_O_2_S_5_ | C_88_H_102_Cl_4_N_6_O_2_S_5_, 2(CHCl_3_) |
| CCDC number | 2374332 | 2374337 |
| Formula weight | 1512.05 | 1816.60 |
| Temperature/K | 200(2) | 200(2) |
| Wavelength/Å | 0.71073 | 0.71073 |
| Crystal system | Triclinic | Monoclinic |
| Space group | P-1 | C2/c |
| a/Å | 11.2366(9) | 24.410(3) |
| b/Å | 15.3392(12) | 23.914(3) |
| c/Å | 26.054(2) | 34.035(4) |
| α/° | 102.716(3) | 90 |
| β/° | 96.609(3) | 107.103(4) |
| γ/° | 102.402(3) | 90 |
| Volume/Å^3^ | 4215.7(6) | 18989(4) |
| Z | 2 | 8 |
| Density/Mg/m^3^ | 1.191 | 1.271 |
| Absorption coefficient/mm^-1^ | 0.196 | 0.452 |
| F(000) | 1608 | 7616 |
| Crystal size/mm^3^ | 0.284 × 0.124 × 0.059 | 0.535 × 0.191 × 0.138 |
| 𝜃 range for data collection/° | 1.920 to 26.250 | 1.923 to 26.413 |
| Index ranges | -13<=h<=13, -19<=k<=19, -32<=l<=32 | -29<=h<=30, -29<=k<=29, -40<=l<=42 |
| Reflections collected | 33341 | 61961 |
| Independent reflections | 16983 [R(int) = 0.0420] | 19484 [R(int) = 0.0678] |
| Completeness to 𝜃 = 25.242° | 99.9 % | 99.9 % |
| Absorption correction | Semi-empirical from equivalents | Semi-empirical from equivalents |
| Max. and min. transmission | 0.9585 and 0.8721 | 0.9583 and 0.7659 |
| Refinement method | Full-matrix least-squares on F^2^ | Full-matrix least-squares on F^2^ |
| Data/restraints/parameters | 16983 / 281 / 808 | 19484 / 596 / 1031 |
| Goodness-of-fit on F^2^ | 1.706 | 1.306 |
| Final R indices [I>2sigma(I)] | R1 = 0.1697, wR2 = 0.4509 | R1 = 0.1375, wR2 = 0.3758 |
| R indices (all data) | R1 = 0.2443, wR2 = 0.5078 | R1 = 0.2282, wR2 = 0.4261 |
| Extinction coefficient | n/a | n/a |
| Largest diff. peak and hole/e.Å^-3^ | 1.820 and -1.182 | 1.211 and -1.033 |

# Photovoltaic device fabrication and characterization

The fabrication of the inverted devices follows the procedures: ITO-coated glass substrates (Sanyo, Japan; 6.4 Ω/sq) were subjected to sequential cleaning in an ultrasonic bath using detergent, deionized (DI) water, acetone, and isopropyl alcohol, each for a duration of 20 minutes. Subsequently, the ITO surface was treated with oxygen plasma using a Harrick Plasma PDC32G system for 5 minutes. The ZnO layer was fabricated using the sol–gel ZnO precursor solution, which was prepared by mixing zinc acetate (3.15 g), ethanolamine (0.9 mL), and 2-methoxyethanol (29.1 mL) at 25 °C for 3 days under continuous stirring. The resulting precursor solution was spin-coated onto the pre-treated ITO-coated glass substrate and subsequently baked in air at 170 °C for 20 minutes. A solution of PM6:CT-F and PM6:CT-Cl in chloroform (16 mg/mL) was prepared by mixing in a weight ratio of 1:1.5 and stirring the solution for 1 hour at 60 °C. The resulting solution was spin-coated onto pre-prepared ZnO/ITO substrates to form active layers. Subsequently, the substrates were thermally annealed at 130 °C for 10 minutes in a glovebox. Finally, a 3 nm-thick MoO_3_ layer and a 100 nm-thick silver anode were deposited by thermal evaporation under a vacuum pressure of less than 10^−6^ torr. The photoelectronic properties of the devices, including the current density-voltage (*J*–*V*), photocurrent density-effective voltage (*J*_ph_–*V*_eff_), short-circuit current density-light intensity (*J*_SC_–light intensity), and open-circuit voltage-light intensity (*V*_OC_–light intensity) curves, were characterized using a computer-controlled Keithley 2400 source measurement system coupled with an AAA-class solar simulator (Enli Technology Co., Ltd., Taiwan). The simulator enables precise control of light intensity ranging from 10 to 100 mW cm^-2^.

**Table S2.** Summary of binary device parameters based on previous NIR NFAs (beyond 1000 nm) reported in the literature (17 papers) and compared with CT-F in this work.

| Year | Acceptor | Donor | λ_onset_ (nm) | *V*_oc_ | *J*_sc_ | FF (%) | PCE | Ref |
| --- | --- | --- | --- | --- | --- | --- | --- | --- |
| 2017 | IEICO-4Cl | PTB7-Th | 1010 | 0.727 | 22.8 | 62.0 | 10.3 | [6] |
| 2017 | IEICO-4F | PBDTTT-EFT | 1000 | 0.739 | 22.8 | 59.4 | 10.0 | [7] |
| 2018 | 6TIC-4F | PBDB-T | 1000 | 0.723 | 23.0 | 67 | 11.14 | [8] |
| 2018 | COTIC-4F | PTB7-Th | 1127 | 0.56 | 20.3 | 56.3 | 7.4 | [9] |
| 2018 | SiOTIC-4F | PTB7-Th | 1060 | 0.65 | 21.6 | 61.4 | 9.0 | [9] |
| 2018 | DTPC-DFIC | PTB7-Th | 1021 | 0.76 | 21.92 | 61.3 | 10.21 | [10] |
| 2019 | CO1-4F | PTB7-Th | 1033 | 0.64 | 24.8 | 64 | 10.2 | [11] |
| 2019 | CO*i*8DFIC | PTB7-Th | 1050 | 0.69 | 27.3 | 71 | 13.8 | [12] |
| 2019 | FUIC | PTB7-Th | 1020 | 0.692 | 22.9 | 70.6 | 11.2 | [13] |
| 2019 | IXIC-4Cl | PM7 | 1000 | 0.795 | 21.6 | 69.9 | 12.01 | [14] |
| 2020 | H3 | PBDB-T | 1016 | 0.757 | 25.84 | 70 | 13.75 | [15] |
| 2021 | BTP-2V-2F | PM6 | 1020 | 0.66 | 26.16 | 64 | 11.22 | [16] |
| 2021 | BTPV-4F | PTB7-Th | 1021 | 0.65 | 28.3 | 65.9 | 12.1 | [17] |
| 2021 | PDTTIC-4F | PM6 | 1030 | 0.62 | 25.90 | 0.67 | 10.70 | [18] |
| 2022 | ATT-9 | PTB7-Th | 1040 | 0.663 | 30.0 | 0.672 | 13.35 | [19] |
| 2022 | DO-4F | PBDB-T | 1000 | 0.75 | 25.9 | 70.0 | 13.6 | [20] |
| 2022 | QO-4F | PBDB-T | 1050 | 0.73 | 10.5 | 61.3 | 4.7 | [20] |
| 2023 | BTPSV-4F | PTB7-Th | 1021 | 0.66 | 28.4 | 69.5 | 13.0 | [21] |
| 2023 | BTPSeV-4F | PTB7-Th | 1061 | 0.66 | 30.1 | 71.4 | 14.2 | [21] |
| 2023 | O6T-4F | PTB7-Th | 1050 | 0.673 | 22.72 | 67.95 | 11.69 | [22] |
| - | CT-F | PM6 | 1002 | 0.79 | 24.61 | 70.9 | 13.74 | This work |

1. **Charge recombination analysis**

To investigate the charge recombination properties, the relationship between *J*_sc_ and light intensity (*P*_light_) was measured. The parameter α in the formula *J*_sc_ ∝ *P*_light_^α^ represents bimolecular recombination. The linear relationship between *V*_oc_ and *P*_light_ was also evaluated by the equation *V*_oc_ ∝ n(kT/q)ln(*P*_light_) where n, q, T, and k, represent the ideality factor, elementary charge, temperature (in Kelvin), and Boltzmann constant, respectively. A value of n = 2 indicates that trap-assisted recombination dominates in the devices, while n~1 suggests less trap-assisted recombination.

1. **Exciton dissociation and collection**

The exciton dissociation probability (*P*_diss_) and charge collection efficiency (*P*_coll_) were systemically studied by measuring photocurrent density (*J*_ph_ = *J*_L_-*J*_D_) versus effective voltage (*V*_eff_ = *V*_0_-*V*_app_), where *J*_L_ and *J*_D_ is the current in the light and in the dark, *V*_0_ is the effective voltage when the *J*_ph_ = 0 V, and *V*_app_ is the applied bias voltage. The *P*_diss_ and *P*_coll_ can be estimated by the formula *J*_sc_/ *J*_sat_ and *J*_mpp_/ *J*_sat_, where *J*_sat_ is the saturation photocurrent, *J*_sc_ and *J*_mpp_ represent photocurrent density under short circuit condition and maximum power point, respectively.

1. **Transient photocurrent (TPC) and transient photovoltage (TPV)**

The comprehensive characterization platform Paios (Fluxim AG) was utilized to evaluate the optoelectronic properties of device, encompassing TPC and TPV measurements. The charge extraction time and carrier lifetime can be extracted by fitting the TPC and TPV curves using an exponential decay function.

**
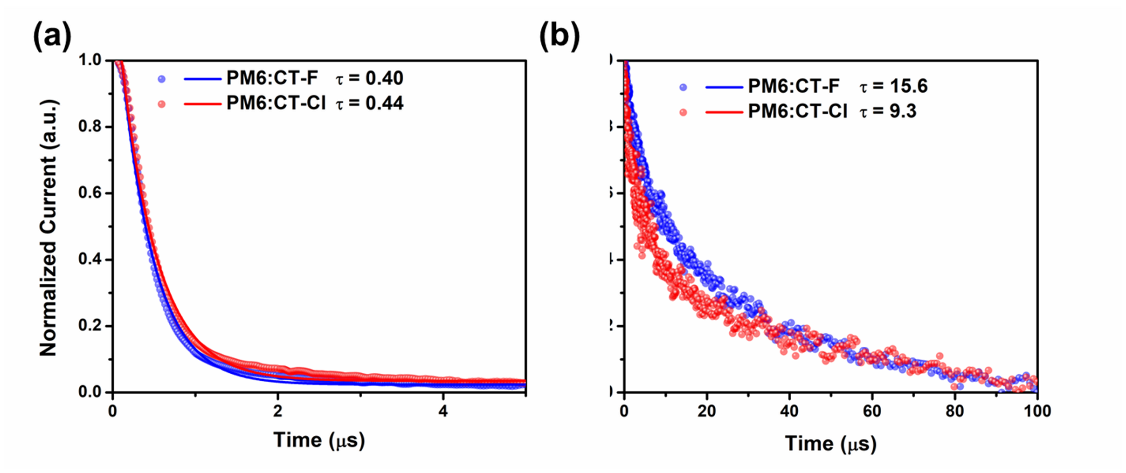
**

**Figure S4.** (a) TPC decay curves, (b) TPV decay curves based on PM6:CT-F and PM6:CT-Cl.

# The photoluminescence (PL) quenching characteristics

CT-F, CT-Cl, PM6, PM6:CT-F and PM6:CT-Cl films were formed by spin-coating on the clean silicon dioxide substrates. PL spectra were obtained from a Edinburgh FLS1000 fluorescence spectrophotometer.

**
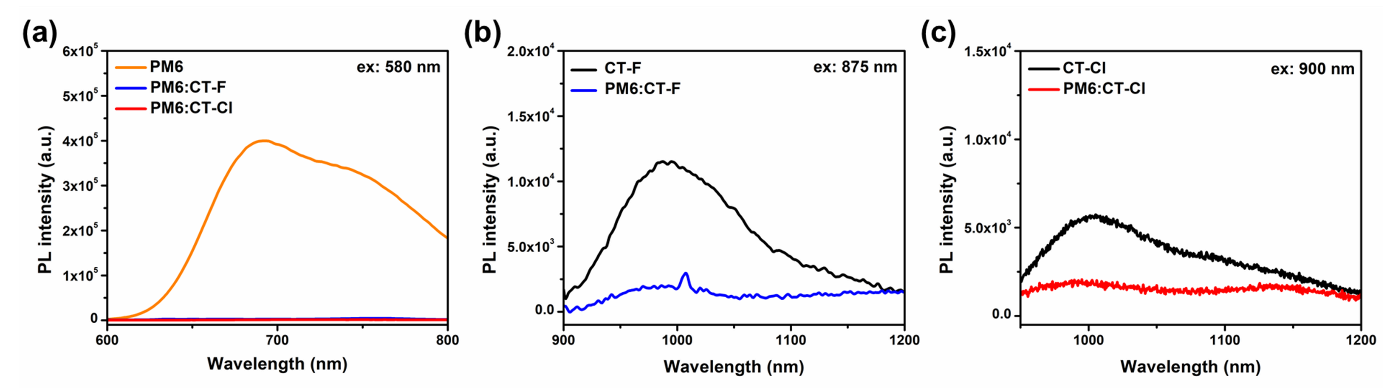
**

**Figure S5.** Photoluminescence spectra of (a) PM6, PM6:CT-F and PM6:CT-Cl films excited at 5 nm; (b) CT-F and PM6:CT-F films excited at 8 nm, (c) CT-Cl and PM6:CT-Cl films excited at 8 nm, and QY is the quenching yield of blending film.

Ultra-pure water and ethylene glycol (EG) are used as wetting liquid.

# Energy loss analysis

Fourier-transform photocurrent spectroscopy-external quantum efficiency (FTPS-EQE) and electroluminescence (EL) spectra were employed to investigate the energy loss (*E*_loss_) mechanisms. The charge transfer state energy (*E*_CT_) was determined by fitting the FTPS-EQE and EL spectra. The non-radiative energy loss (Δ*E*_non-rad_) was calculated from the external quantum efficiency of electroluminescence (EQE_EL_) using the equation:

$$\Delta E_{non-rad}=-\frac{k_{B}T}{q}ln(\mathrm{EQE}_{\mathrm{EL}})$$

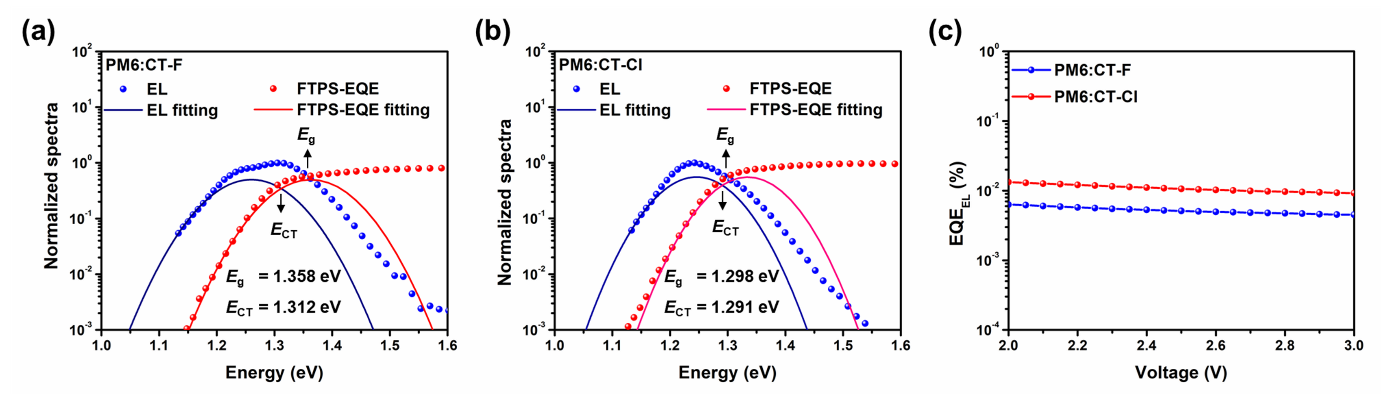


**Figure S6.** The normalized and fitted FTPS-EQE and EL curves for the devices based on (a) PM6:CT-F and (b) PM6:CT-Cl. (c) The EQE_EL_ spectra of the OPVs.

**Table S3.** The detailed energy loss parameters of the OPVs.

| Active layer | *E*_gap_  [eV] | q*V*_OC_  [eV] | *E*_loss_  [eV] | *E*_CT_  [eV] | Δ*E*_CT_  [eV] | Δ*E*_rad_  [eV] | Δ*E*_non-rad_  [eV] | EQE_EL_ |
| --- | --- | --- | --- | --- | --- | --- | --- | --- |
| PM6:CT-F | 1.358 | 0.78 | 0.578 | 1.312 | 0.046 | 0.279 | 0.253 | 5.23 ×10^-5^ |
| PM6:CT-Cl | 1.298 | 0.78 | 0.518 | 1.291 | 0.007 | 0.277 | 0.234 | 1.09 ×10^-4^ |

**Table S4.** Structural parameters derived from the GIWAXS patterns of NFAs and their blends with PM6, including the out-of-plane and in-plane reflection peaks centered at *q*_z_ and *q*_xy_, with the corresponding *d*-spacing (*d*_π_, *d*_l_) and the coherence length (*L*_c_) deduced from the full-width at half-maximum (FWHM) using the Scherrer equation with a shape factor of 0.9.

| materials | in-plane reflection peak | | | | out-of-plane reflection peak | | | |
| --- | --- | --- | --- | --- | --- | --- | --- | --- |
|  | *q*_xy_ [Å^-1^] | *d*_l_ [Å] | FWHM [Å^-1^] | *L*_c_ [Å] | *q*_z_ [Å^-1^] | *d*_π_ [Å] | FWHM [Å^-1^] | *L*_c_ [Å] |
| CT-F | 0.353 | 17.80 | 0.197 | 28.76 | 1.781 | 3.526 | 0.327 | 17.28 |
| CT-Cl | 0.367 | 17.11 | 0.187 | 30.15 | 1.778 | 3.532 | 0.283 | 19.97 |
| PM6:CT-F^[a]^ | 0.305 | 20.59 | 0.133 | 42.50 | 1.760 | 3.569 | 0.290 | 19.49 |
| PM6:CT-Cl^[a]^ | 0.298 | 21.07 | 0.143 | 39.52 | 1.738 | 3.613 | 0.280 | 20.19 |

[a] Thermal annealing at 130 °C for 10 minutes.

# Fabrication and characterization of OPD devices

The OPD devices employed the same ITO/ZnO/PM6:NFAs/MoO_3_/Ag structure as the OPV devices. However, to optimize the OPD performance, the thickness of the active layer was increased to suppress charge injection. The active layer precursor solution had a concentration of 26 mg/mL (in CF) to achieve an active layer thickness of approximately 220-250 nm.

A programmable power controller (Keithley Model 2636A) was employed to measure the dark current density of the OPDs under dark conditions. The EQE spectra and responsivities of the OPDs were measured using a photoresponse measurement system (QE-R3011, Enli Technology Co., Ltd.). Monochromatic light beams from a calibrated commercial light source (Newport, TLS-300XR) were chopped at a frequency of 250 Hz by an optical chopper system before illumination of the devices. The photocurrent response was recorded using a lock-in amplifier (Signal Recovery 7225). The power density of the LED light source (Thorlabs, M530L4) was corrected using a Newport UV 818-L instrument. Subsequently, the light was passed through a motorized filter wheel (Thorlabs, FW102CNEB) to illuminate the area of the OPD device for LDR measurement. The response of the organic photodiode (OPD) device was modulated by a light beam generated from a commercial LED (Thorlabs, emission wavelength: 530 nm, flux density: 1 mW/cm²). The photocurrent response was then passed a low-noise current preamplifier (Ametek model 5182) with no bandwidth filter. Finally, the signal was displayed and recorded by a 2.5-GHz oscilloscope (Teledyne LeCroy WaveRunner 625Zi) for measurement of the frequency response. The rise and fall times of the photocurrent response were measured using a commercial LED (Thorlabs, emission wavelength: 530 or 780 nm, luminous flux density: 1.00 mW cm^-2^) with 1-ms frequency pulses.

**
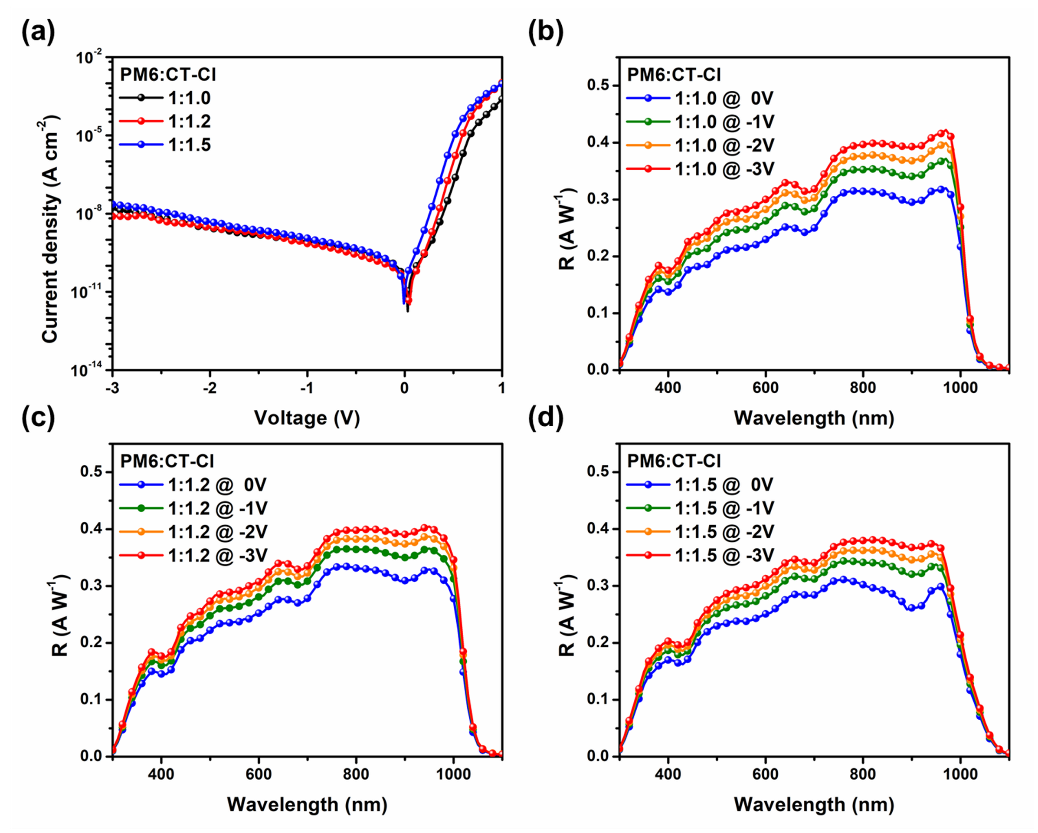
**

Figure S7. (a) Characteristic *J*-*V* curves of PM6:CT-Cl in the dark with 1:1, 1:1.2 and 1:1.5 donor-to-acceptor (D:A) blend ratios. The R curves of (b) 1:1 (c) 1:1.2 and (d) 1:1.5 blend weight ratio from 0 V to -3 V, respectively, and (d) *D*_sh_* curves of the PM6:CT-F-based OPDs from 0 V to −3 V.

**Table S5.** Photoresponse parameters of the PM6:CT-Cl-based OPDs with varying donor-to-acceptor (D:A) blend ratios under reverse bias.

| Material | D:A ratios | bias [V] | *J*_d_ (A cm^−2^) | R (A·W^-1^)^[a]^ | *D*_sh_* (Jones)^[a]^ |
| --- | --- | --- | --- | --- | --- |
| PM6:CT-Cl | 1:1.0 | -1 | 8.5 × 10^−10^ | 0.36 | 2.2 × 10^13^ |
|  |  | -3 | 1.7 × 10^−8^ | 0.41 | 5.6 × 10^12^ |
|  | 1:1.2 | -1 | 7.1 × 10^−10^ | 0.37 | 2.4 × 10^13^ |
|  |  | -3 | 7.8 × 10^−9^ | 0.40 | 8.1 × 10^12^ |
|  | 1:1.5 | -1 | 1.2 × 10^−9^ | 0.33 | 1.8 × 10^13^ |
|  |  | -3 | 2.3 × 10^−8^ | 0.37 | 4.3 × 10^12^ |

[a] R and *D***_sh_*** at 950 nm.

# Secondary ion mass spectrometry (SIMS) measurement

Secondary ion mass spectrometry (SIMS) was utilized to examine the vertical composition of the PM6:CT-F and PM6:CT-Cl-based OPD devices by analyzing the distribution of cyano (CN) functional groups.

**

**

**Figure S8.** Plot of CN functional group intensity versus depth for CT-F and CT-Cl-based OPD devices by Secondary Ion Mass Spectrometry (SIMS).


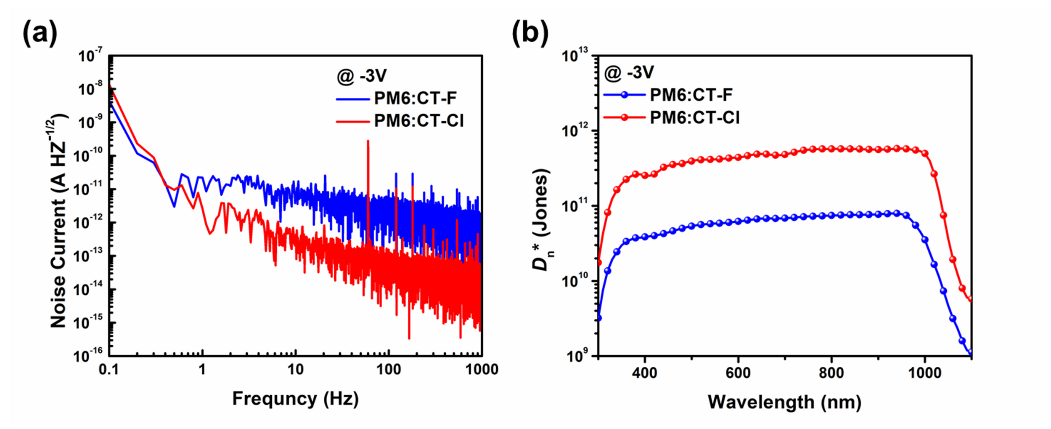


**Figure S9**. (a) Actual noise of PM6:CT-Cl- and PM6:CT-F-based OPDs and (b) corresponding values of *D*_n_* determined from measured noise currents.


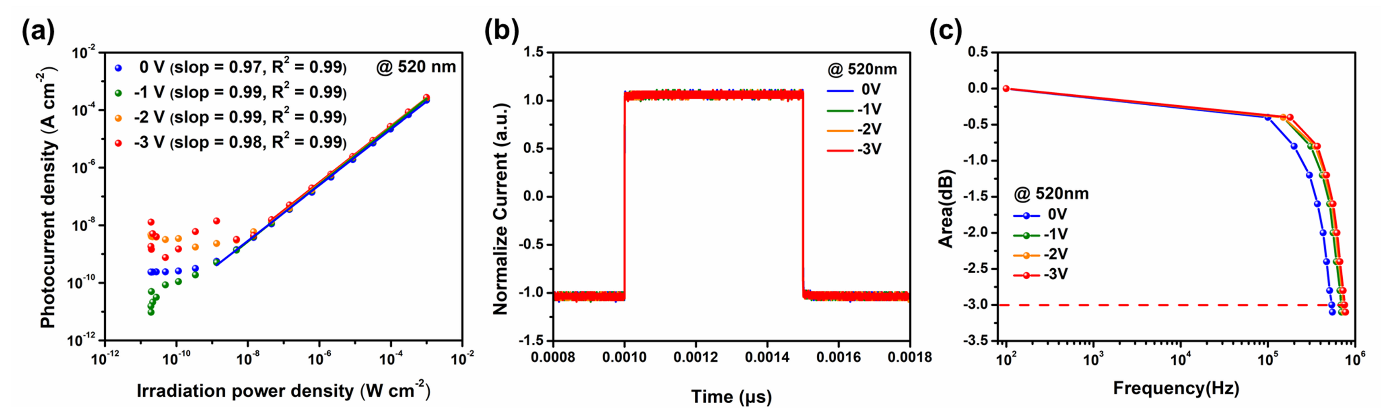


Figure S10. (a) LDR curves, (b) the rise/fall times, (c) the cut-off frequencies (*f*_−3dB_) using 520 nm light source of the PM6:CT-Cl-based OPDs.





**Figure S11.** EIS analysis of the PM6:CT-Cl-based OPD at various biases.

**Table S6.** Photoresponse performance of the CT-Cl-based OPDs device using 520 nm and 780 nm light sources.

| Material | LED  light sources | bias | LDRs (dB) | τ_r_/τ_f_ (μs) | *f*_−3dB_ (kHz) |
| --- | --- | --- | --- | --- | --- |
| PM6:CT-Cl | 520 nm | 0 V | 117.5 | 0.32/0.18 | 550 |
|  |  | -1 V | 106.2 | 0.29/0.11 | 700 |
|  |  | -2 V | 96.6 | 0.27/0.11 | 750 |
|  |  | -3 V | 86.7 | 0.25/0.10 | 780 |
|  | 780 nm | 0 V | 124.0 | 0.35/0.21 | 420 |
|  |  | -1 V | 115.9 | 0.33/0.11 | 590 |
|  |  | -2 V | 98.8 | 0.30/0.11 | 630 |
|  |  | -3 V | 83.8 | 0.29/0.10 | 650 |

**Table S7.** Summary of OPD parameters and performances based on previous NIR NFAs (beyond 1000 nm) reported in the literature (9 papers) and compared with CT-Cl in this work.

| Photosensitive  materials | λ_onset_ of  Response  (nm) | *J*_d_  (nA cm^-2^)  [bias] | R  (A W^-1^) [λ, bias] | *D*_sh_*  (Jones)  [λ, bias] | LDR  (dB)  [λ, bias] | ƒ_-3dB_  (kHz)  [λ, bias] | Rise/fall time  (μs)  [λ, bias] | Ref. |
| --- | --- | --- | --- | --- | --- | --- | --- | --- |
| PM6:CT-Cl | 1042 | 0.71/1.4  [-1 V/-3 V] | 0.37/0.40  [950 nm, -1 V/-3 V] | 1.3 × 10^14^ / 2.4 x 10^13^ / 8.1 x 10^12^  [950 nm, 0 v/-1 V/-2 V] | 115.9/83.8 [LED 780 nm, -1 V/-3 V] | 590/650  [LED 780 nm, -1 V/-3 V] | 0.33/0.11 [LED 780 nm, -1 V] | This work |
| PBDB-T:DO-4F | 1000 | 0.86  [0 V] | 0.50  [850 nm, 0 V] | 3.05 x 10^13^  [850 nm, 0 V] | - | - | - | [20] |
| PTB7-Th:PCBM:O6T-4F | 1050 | 5.7 × 10^−6^ (noise current) | 0.52  [850 nm, 0 V] | 1.10 x 10^14^  [850 nm, 0 V] | 144 [LED 808 nm, 0 V] | 370  [0 V] | 0.537/0.489 [0 V] | [22] |
| PBDT-TT:DPPSe-4Cl:Y6 | ~1060 ^[^**^a]^** | 1.0 / ~10 ^[^**^a]^**  [-0.1 V/-2 V] | 0.18  [940 nm, -0.1 V] | 1.0 x 10^13^  [950 nm, -0.1 V] | 85 | 33 | 2/29 | [23] |
| PTB7-Th  :TTD(DTC-2FIC)_2_ | ~1350 ^[^**^a]^** | 202 / ~1000 ^[^**^a]^**  [-0.5 V/-2 V] | 0.095  [1100 nm, -0.5 V] | 1.1 x 10^13^  [1100 nm, -0.5 V] ^[b]^ | 60.8 [LED 650 nm, -0.5 V] | - | 223.46/11.71  [LED 650 nm, -0.5 V] | [24] |
| PTB7-Th:COT-Oct | ~1110 ^[^**^a]^** | 8.18  [-0.5 V] | 0.24  [1000 nm, -0.5 V] | 1.49 x 10^12^  [1000 nm, -0.5 V] | - | 72  [LED 980 nm, -0.5 V] | - | [25] |
| PCE10:FB-C6 | 1150 | 45.9/~1000 ^[^**^a]^**  [-0.1 V/-2 V] | 0.29  [970 nm, -0.1 V] | 2.39 x 10^12^  [970 nm, -0.1 V] | 121  [940 nm] | 7.8 [940 nm] | - | [26] |
| PCE10:YZ1 | 1050 | 0.053/~100 ^[^**^a]^**  [0 V/-1 V] | 0.27  [1000 nm, 0 V] | 9.24 x 10^13^  [1000 nm, 0 V] | 127 [905 nm] | 580  [905 nm] | 0.976/0.600 [905 nm, 0 V] | [27] |
| PTB7-Th:  COTIC-4F:PC71BM | ~1140 ^[^**^a]^** | 0.22/110  [0 V/-1 V] | 0.48/0.53  [940 nm, 0 V/-1 V] | 3.4 x 10^13^  [940 nm, 0 V] | 123  [660 nm, -1V] | 1140 [940 nm] | - | [28] |
| PTB7-Th:YOR1 | 1000 | 0.158/~10 ^[^**^a]^**  [-0.1 V/-2 V] | 0.141  [950 nm] | 1.98 x 10^13^  [950 nm, -0.1 V] | 137  [950 nm, -0.1 V] | 91 [950 nm] | - | [29] |
| PBDB-T: FM2 | 1040 | 6.45/32.2  [-0.5 V/-2 V] | 0.455  [880 nm, -0.5 V] | 1.01 x 10^13^ / 4.48 x 10^12^  [880 nm, -0.5 V/-2 V] | - | 7.6 [0 V] | 49/14 [0 V] | [30] |
| PTB7-Th:tSPT-4F | 1100 | 0.452/~1 ^[^**^a]^**  [-0.1 V/-2 V] | 0.40  [1010 nm, 0 V] | 1.25 x 10^13^  [1010 nm, -0.1 V] ^[b]^ | 130  [940 nm, -0.1 V] | 76  [940 nm, -0.1 V] | - | [31] |
| PM6:ABTPV-S | ~1000 | 0.11/2470  [0 V/-2 V] | 0.39/0.41  [0 V, 840 nm/-2 V, 860 nm] | 3.43 x 10^12^  [840 nm, 0 V] | 140 [LED 785 nm, 0 V] | 210  [0 V] | 1.07/0.71 [0 V] | [32] |
| PM6:PDTTYM | 1020 | 3.88  [0 V] | 0.48  [900 nm, 0 V] | 1.31 × 10^13^  [900 nm, 0 V] | 134  [850 nm, 0 V] | 145  [0 V] | 5.8/8.4 [0 V] | [33] |
| PCE10:BTPV-4F-eC9 | 1050 | 0.465  [0 V] | 0.56  [900 nm, 0 V] | 4.6 × 10^13^  [900 nm, 0 V] | 120  [940 nm] | >100  [940 nm] | 8/7.6 [0 V] | [34] |
| [a] The dark current and responsivity values were obtained from the corresponding figures in the literature. [b] Based on Sn. | | | | | | | |  |

# ^1^H and ^13^C NMR spectra


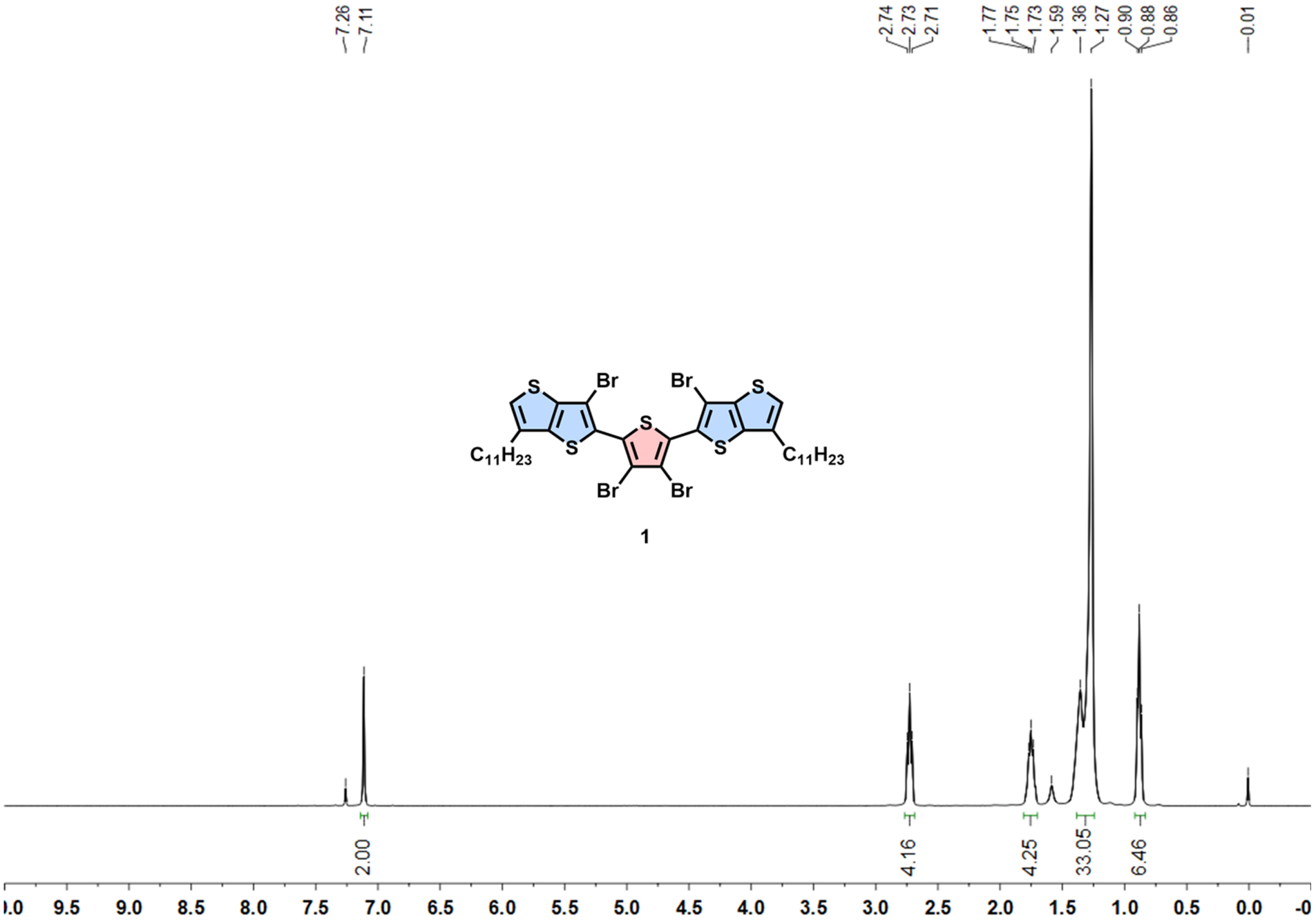


^1^H NMR spectra of compound **1**.


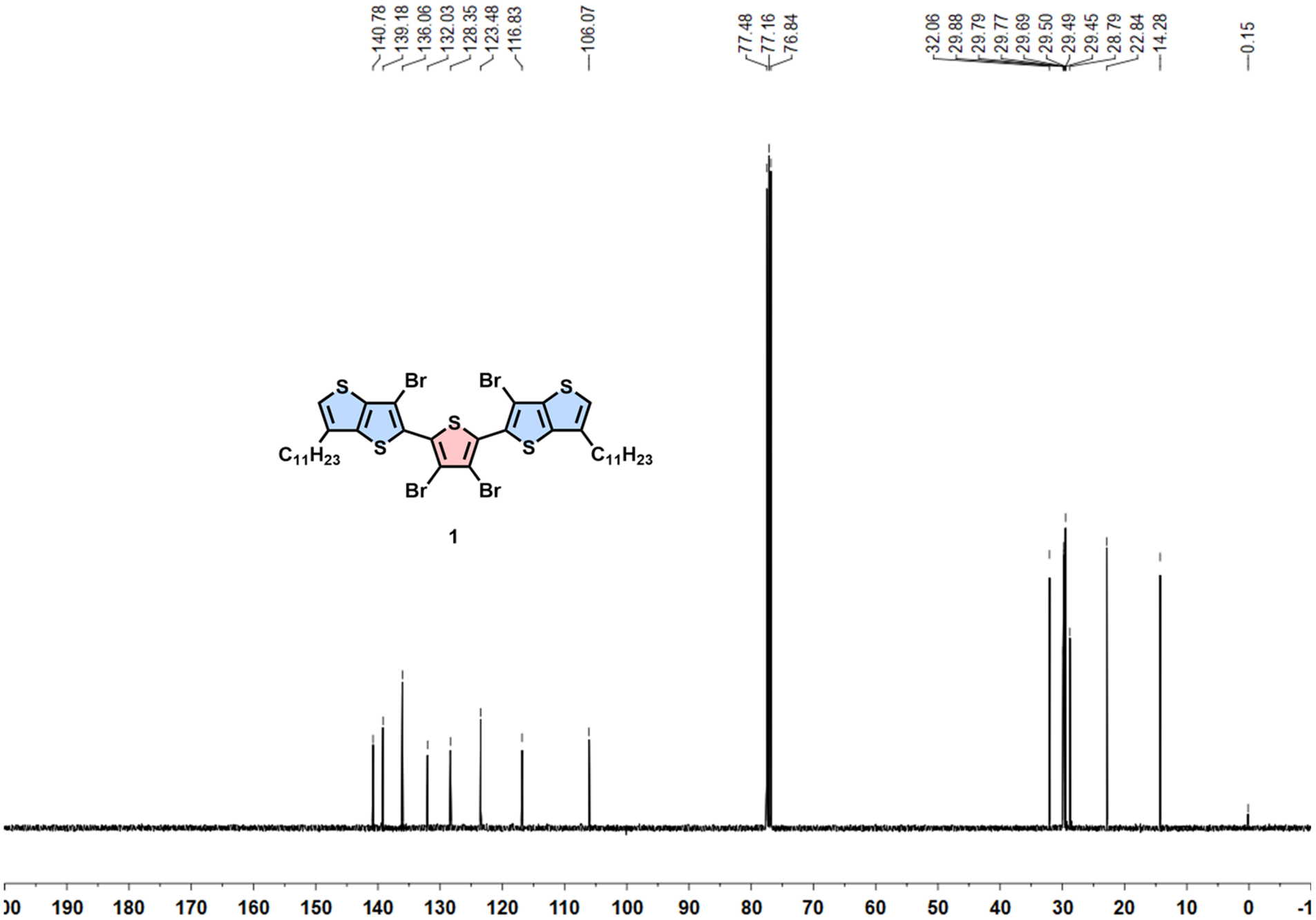


^13^C NMR spectra of compound **1**.


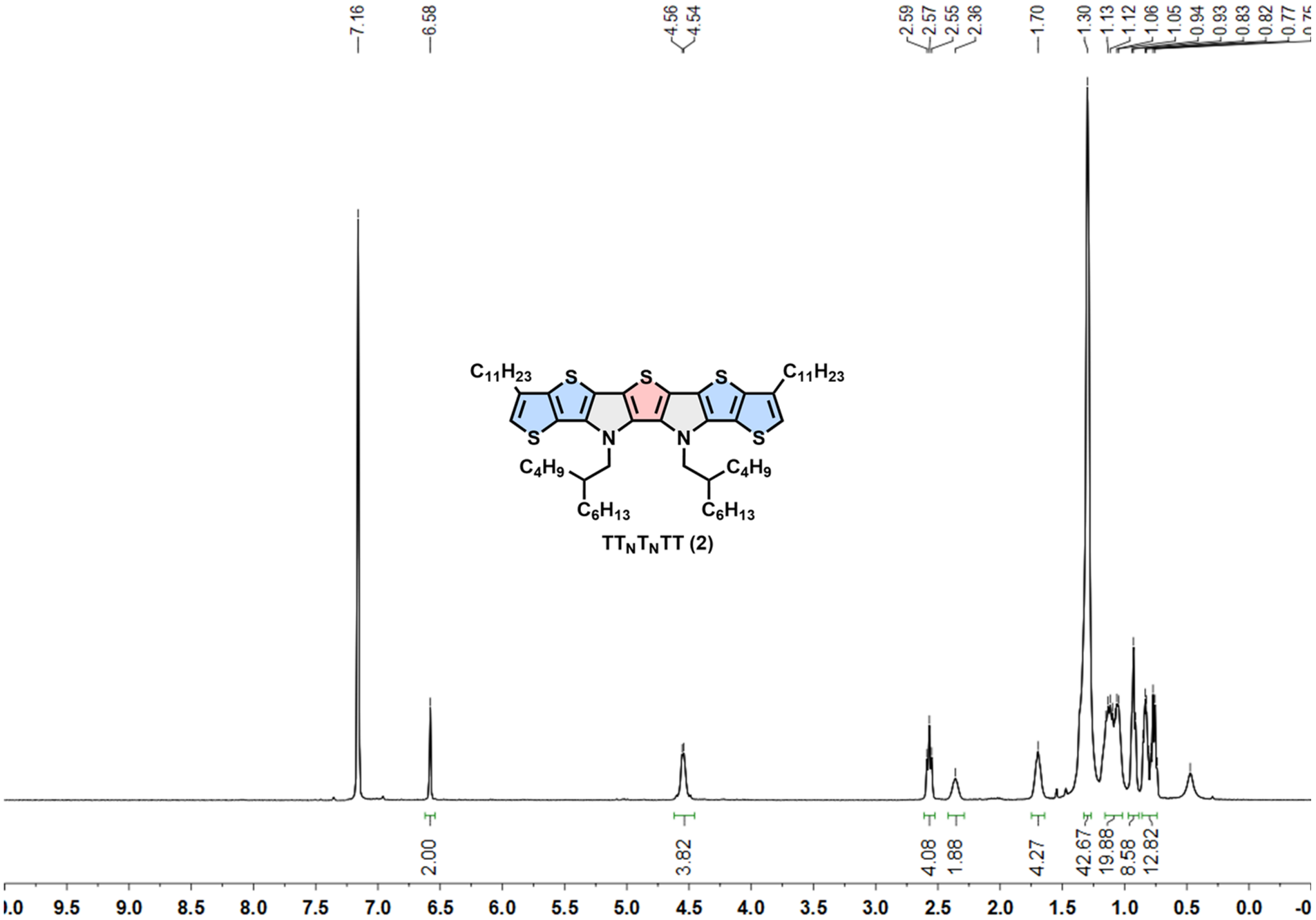


^1^H NMR spectra of compound **TT_N_T_N_TT (2)**.


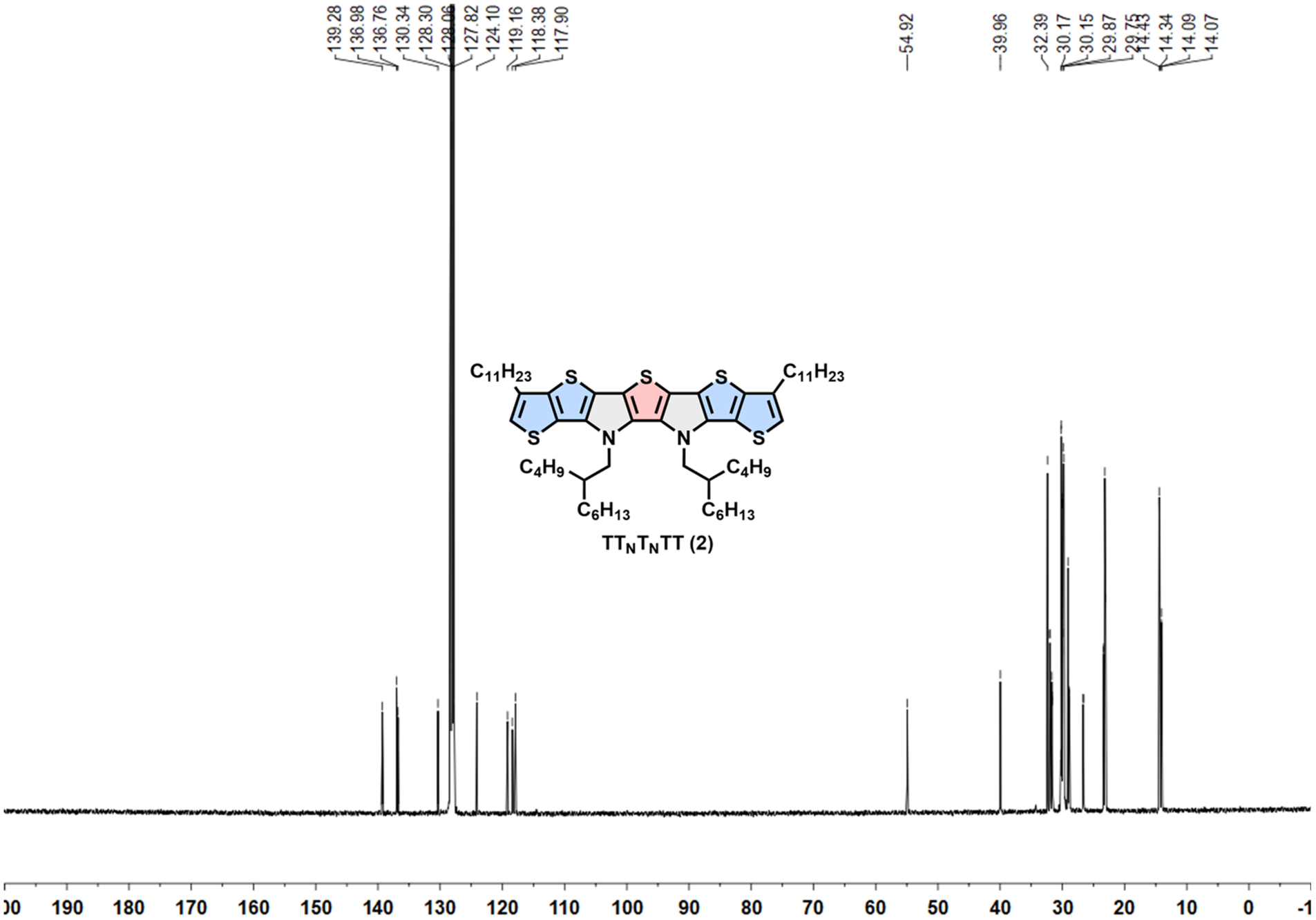


^13^C NMR spectra of compound **TT_N_T_N_TT (2).**

**
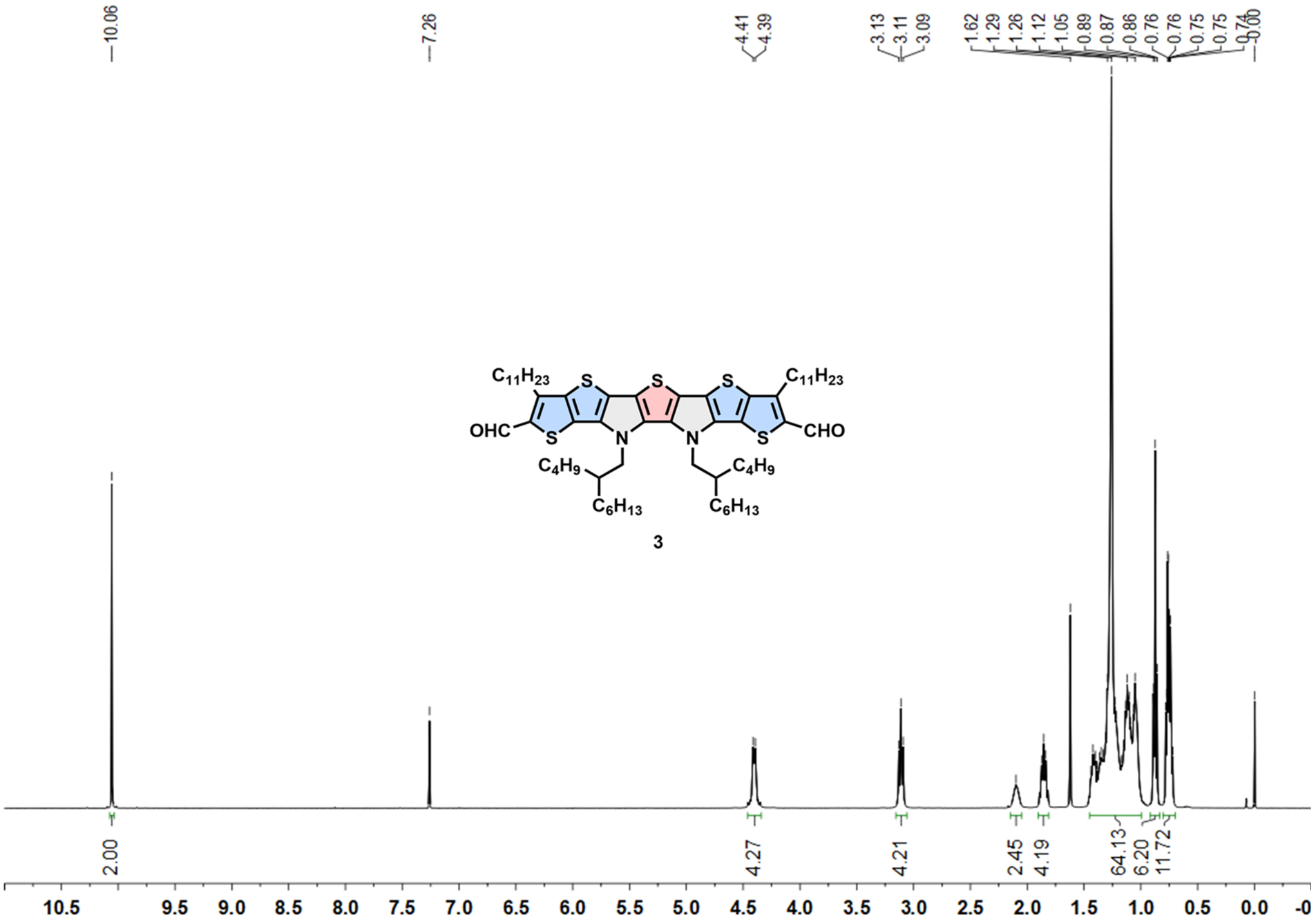
**

^1^H NMR spectra of compound **3**.

**
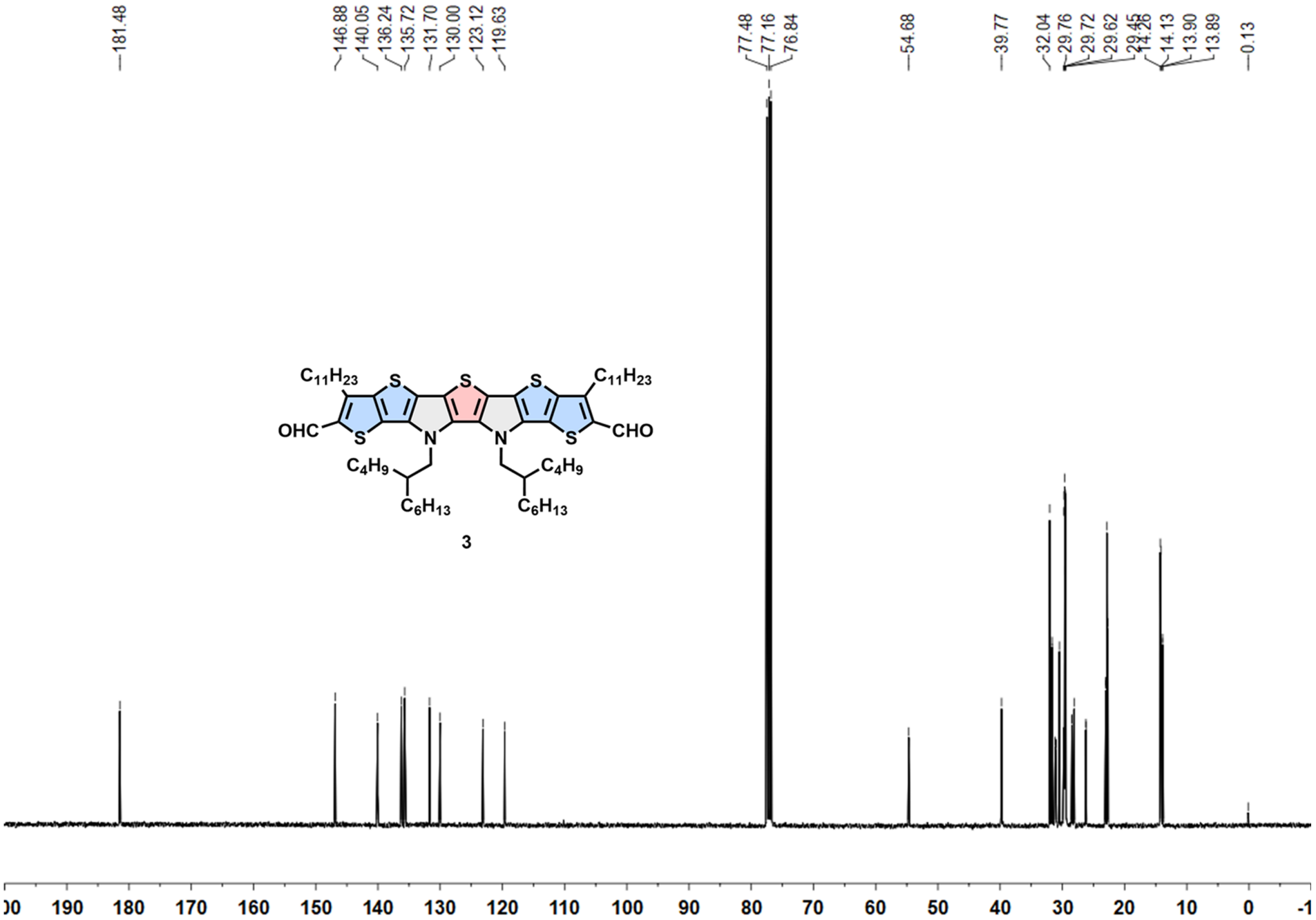
**

^13^C NMR spectra of compound **3**.

**
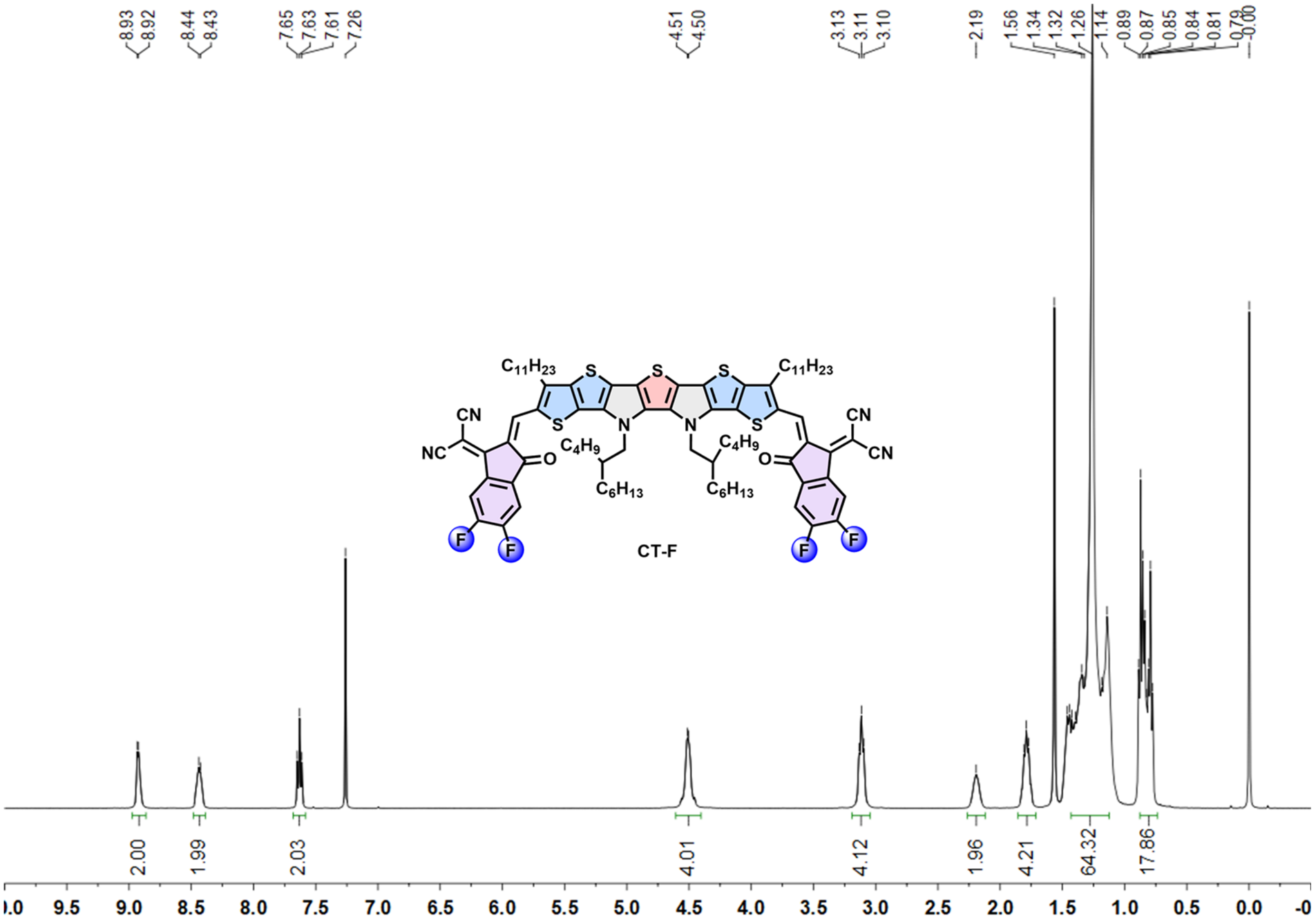
**

^1^H NMR spectra of compound **CT-F**.

**
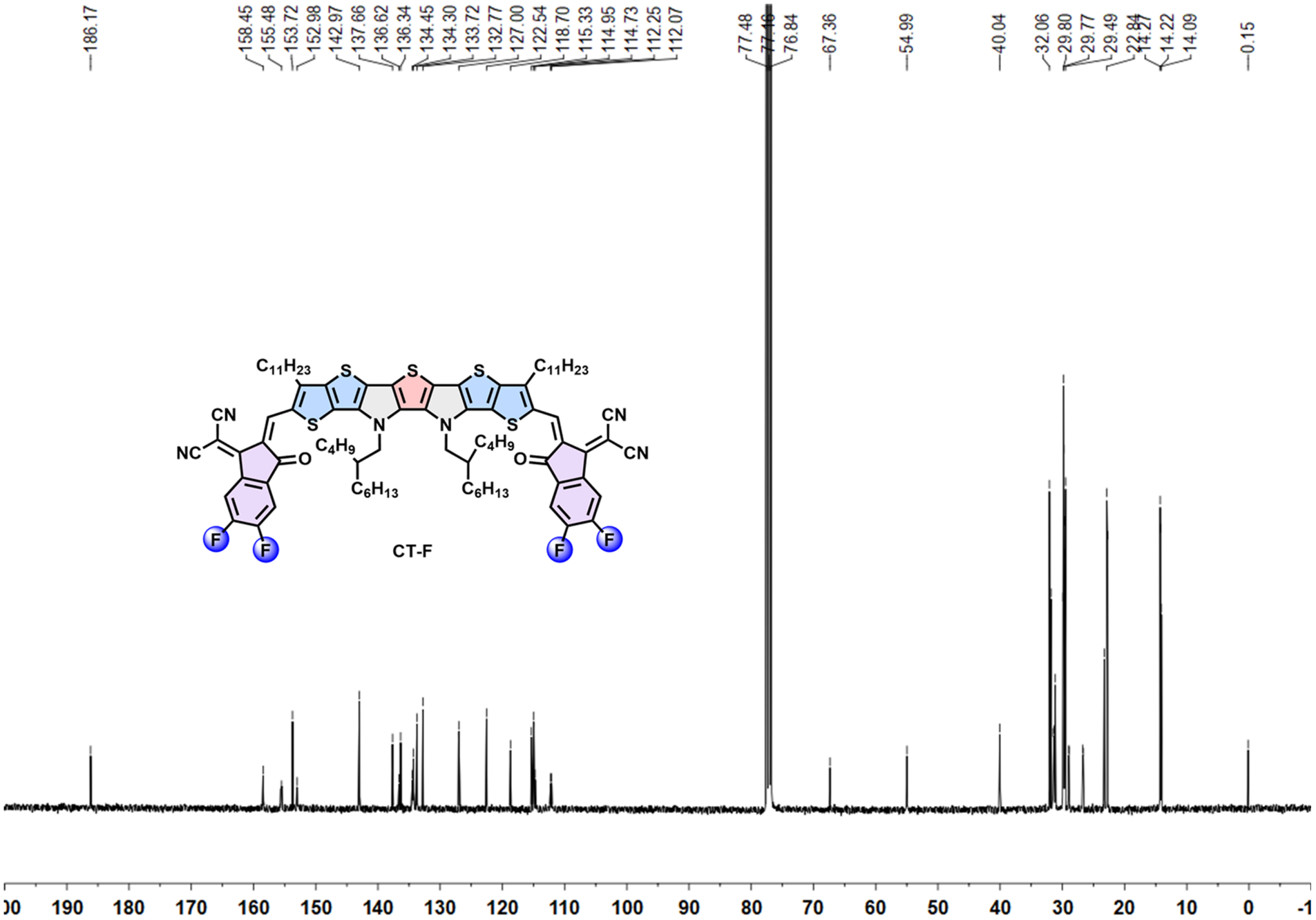
**

^13^C NMR spectra of compound **CT-F**.

**
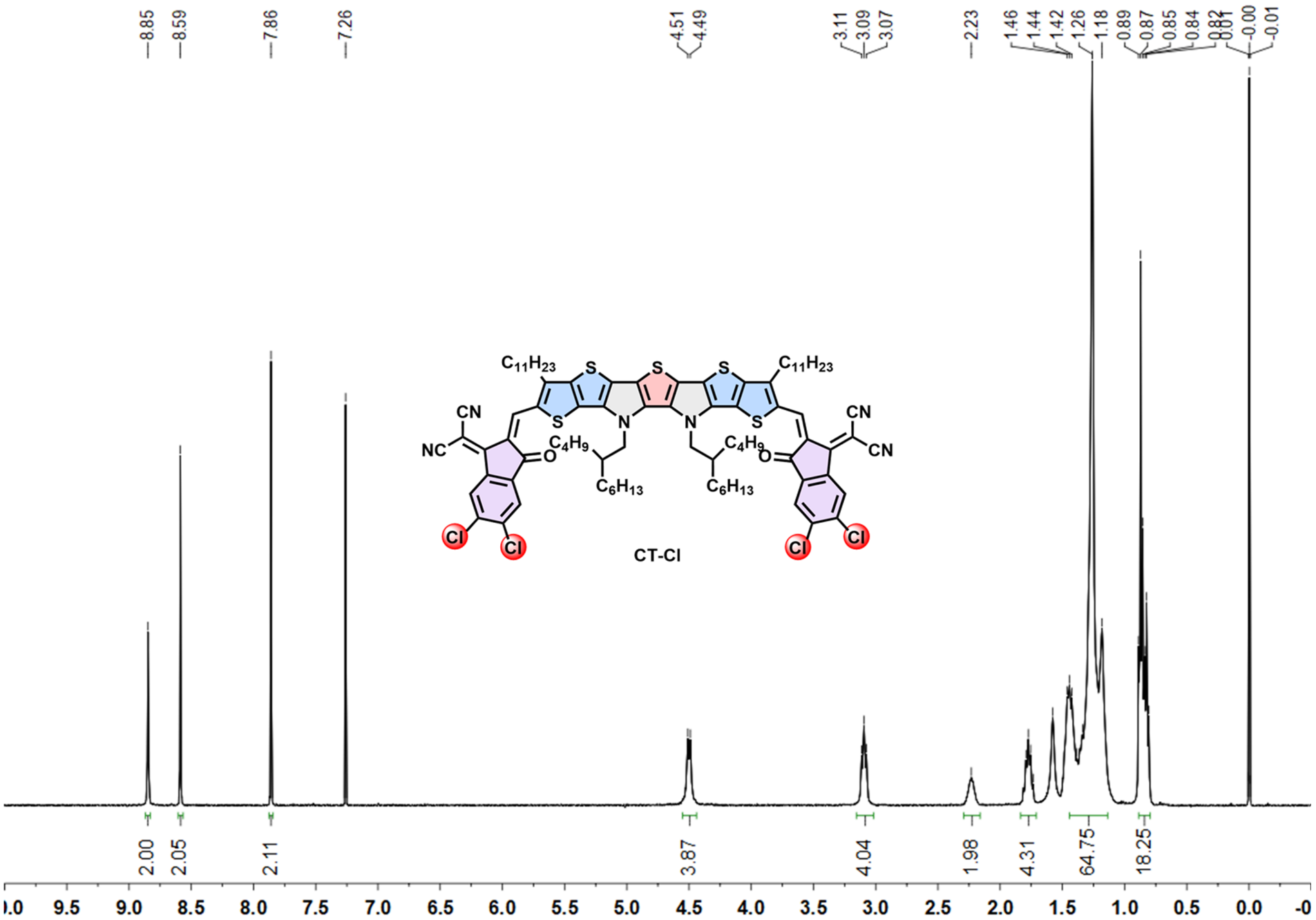
**

^1^H NMR spectra of compound **CT-Cl**.

**
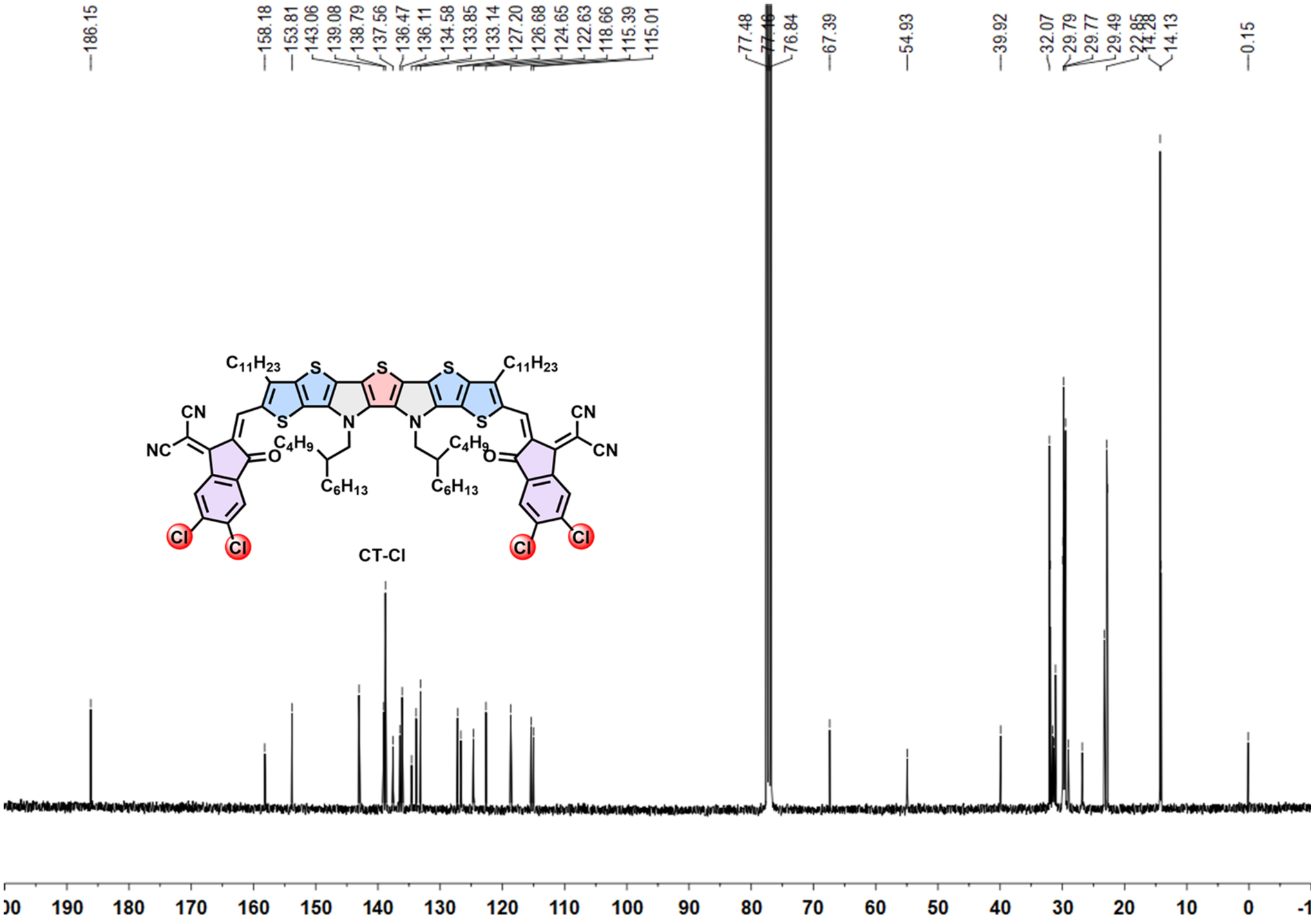
**

^13^C NMR spectra of compound **CT-Cl**.

# References

[1] Y.-J. Xue, Z.-Y. Lai, H.-C. Lu, J.-C. Hong, C.-L. Tsai, C.-L. Huang, K.-H. Huang, C.-F. Lu, Y.-Y. Lai, C.-S. Hsu, J.-M. Lin, J.-W. Chang, S.-Y. Chien, G.-H. Lee, U.-S. Jeng, Y.-J. Cheng, *J. Am. Chem. Soc.* **2024**, *146*, 833-848.

[2] K. SAINT V8.40B; Bruker AXS GmbH, Germany, **2019**.

[3] L. Krause, R. Herbst-Irmer, G. M. Sheldrick, D. Stalke, *J. Appl. Cryst.* **2015**, *48*, 3-10.

[4] G. M. Sheldrick, *Acta Crystallogr., Sect. C: Struct. Chem.* **2015**, *71*, 3-8.

[5] K. APEX4 v.2021.2010-2020; Bruker AXS GmbH, Germany, **2021**.

[6] Y. Cui, C. Yang, H. Yao, J. Zhu, Y. Wang, G. Jia, F. Gao, J. Hou, *Adv. Mater.* **2017**, *29*, 1703080.

[7] H. Yao, Y. Cui, R. Yu, B. Gao, H. Zhang, J. Hou, *Angew. Chem. Int. Ed.* **2017**, *56*, 3045-3049.

[8] X. Shi, X. Liao, K. Gao, L. Zuo, J. Chen, J. Zhao, F. Liu, Y. Chen, Alex. K.-Y. Jen, *Adv. Funct. Mater.* **2018**, *28*, 1802324.

[9] J. Lee, S. J. Ko, M. Seifrid, H. Lee, B. R. Luginbuhl, A. Karki, M. Ford, K. Rosenthal, K. Cho, T. Q. Nguyen, G. C. Bazan, *Adv. Energy Mater.* **2018**, *8*, 1801212.

[10] Z. Yao, X. Liao, K. Gao, F. Lin, X. Xu, X. Shi, L. Zuo, F. Liu, Y. Chen, Alex. K.-Y. Jen, *J. Am. Chem. Soc.* **2018**, *140*, 2054-2057.

[11] J. Lee, S.-J. Ko, H. Lee, J. Huang, Z. Zhu, M. Seifrid, J. Vollbrecht, V. V. Brus, A. Karki, H. Wang, K. Cho, T.-Q. Nguyen, G. C. Bazan, *ACS Energy Lett.* **2019**, *4*, 1401-1409.

[12] W. Li, M. Chen, J. Cai, E. L. K. Spooner, H. Zhang, R. S. Gurney, D. Liu, Z. Xiao, D. G. Lidzey, L. Ding, T. Wang, *Joule* **2019**, *3*, 819-833.

[13] T. Li, L. Yang, Y. Xiao, K. Liu, J. Wang, X. Lu, X. Zhan, *J. Mater. Chem. A* **2019**, *7*, 20667-20674.

[14] T. Liu, Z. Luo, Y. Chen, T. Yang, Y. Xiao, G. Zhang, R. Ma, X. Lu, C. Zhan, M. Zhang, C. Yang, Y. Li, J. Yao, H. Yan, *Energy Environ. Sci.* **2019**, *12*, 2529-2536.

[15] C. He, Y. Li, Y. Liu, Y. Li, G. Zhou, S. Li, H. Zhu, X. Lu, F. Zhang, C.-Z. Li, H. Chen, *J. Mater. Chem. A* **2020**, *8*, 18154-18161.

[16] J. Hai, W. Zhao, S. Luo, H. Yu, H. Chen, Z. Lu, L. Li, Y. Zou, H. Yan, *Dyes Pigm.* **2021**, *188*, 109171.

[17] Z. Jia, S. Qin, L. Meng, Q. Ma, I. Angunawela, J. Zhang, X. Li, Y. He, W. Lai, N. Li, H. Ade, C. J. Brabec, Y. Li, *Nat. Commun.* **2021**, *12*, 178.

[18] Y. Chen, Y. Zheng, Y. Jiang, H. Fan, X. Zhu, *J. Am. Chem. Soc.* **2021**, *143*, 4281-4289.

[19] W. Liu, S. Sun, S. Xu, H. Zhang, Y. Zheng, Z. Wei, X. Zhu, *Adv. Mater.* **2022**, *34*, e2200337.

[20] Y. Xu, T. Zhang, H. Yao, J. Wang, P. Bi, J. Hou, *Adv. Electron. Mater.* **2022**, *72*, 388-394.

[21] Z. Jia, Q. Ma, Z. Chen, L. Meng, N. Jain, I. Angunawela, S. Qin, X. Kong, X. Li, Y. M. Yang, H. Zhu, H. Ade, F. Gao, Y. Li, *Nat. Commun.* **2023**, *14*, 1236.

[22] C. Shan, T. Liu, J. Zhou, Y. He, D. Luo, Z. Jiang, Z. Wang, Q. Liu, C. a. Li, F. Zhang, E. Zhou, K. Wang, A. K. K. Kyaw, *J. Chem. Eng.* **2023**, *471*, 144451.

[23] Y. Wang, M. Yang, B. Yin, B. Wu, G. Liu, S. Jeong, Y. Zhang, C. Yang, Z. He, F. Huang, Y. Cao, C. Duan, *ACS Appl. Mater. Interfaces* **2024**, 10.1021/acsami.3c15365.

[24] H. Zhang, R. Mao, L. Yuan, Y. Wang, W. Liu, J. Wang, H. Tai, Y. Jiang, *ACS Appl. Mater. Interfaces* **2024**, *16*, 9088-9097.

[25] K. Cho, J. W. Ha, M. Nam, C. Lee, S. J. You, A. Y. Lee, S. C. Yoon, M. Han, J. H. Kim, S. J. Ko, D. H. Ko, *Adv. Funct. Mater.* **2024**, 10.1002/adfm.202400676, 2400676.

[26] Y. Wang, J. Wang, J. Miao, J. Liu, L. Wang, *CCS Chem.* **2024**, 10.31635/ccschem.024.202403865.

[27] Y. Xia, C. Geng, X. Bi, M. Li, Y. Zhu, Z. Yao, X. Wan, G. Li, Y. Chen, *Adv. Opt. Mater.* **2023**, *12*, 2301518.

[28] Z. Lou, J. Tao, B. Wei, X. Jiang, S. Cheng, Z. Wang, C. Qin, R. Liang, H. Guo, L. Zhu, P. Muller-Buschbaum, H. M. Cheng, X. Xu, *Adv. Sci.* **2023**, *10*, e2304174.

[29] J. W. Ha, A. Y. Lee, H. J. Eun, J. H. Kim, H. Ahn, S. Park, C. Lee, D. W. Seo, J. Heo, S. C. Yoon, S. J. Ko, J. H. Kim, *ACS Nano* **2023**, *17*, 18792-18804.

[30] Y. Zhang, Y. Yu, X. Liu, J. Miao, Y. Han, J. Liu, L. Wang, *Adv. Mater.* **2023**, *35*, e2211714.

[31] L. Shao, L. Hong, Y. Cao, H. Tang, Y. Huang, X. Xia, Y. Bai, M. Dong, X. Zhang, X. Lu, X. Yang, C. Liu, F. Huang, Y. Cao, *Adv. Opt. Mater.* **2023**, *11*, 2202823.

[32] Z. Zhong, X. Liu, L. Li, Z. Han, Y. He, X. Xu, J. Hai, R. Zhu, J. Yu, *Science China Chemistry* **2022**, *66*, 242-250.

[33] Y. Zheng, Y. Chen, Y. Cao, F. Huang, Y. Guo, X. Zhu, *ACS Materials Letters* **2022**, *4*, 882-890.

[34] T. Liu, Z. Jia, Y. Song, N. Yu, Q. Lin, C. Li, Y. Jia, H. Chen, S. Wang, Y. Wei, Y. Lin, F. Huang, Z. Tang, Y. Li, L. Meng, H. Huang, *Adv. Funct. Mater.* **2023**, *33*, 2301167.
